# Failure mechanisms data analysis during tension of additively manufactured Ti-6Al-4V alloy reinforced with nano-zirconia particles: Investigations of the crack path

**DOI:** 10.1016/j.dib.2024.110283

**Published:** 2024-03-01

**Authors:** Benjamin Guennec, Amine Hattal, Kentaro Nagano, Azziz Hocini, Kamilla Mukhtarova, Takahiro Kinoshita, Noriyo Horikawa, Hiroshi Fujiwara, Jenő Gubicza, Madjid Djemaï, Guy Dirras

**Affiliations:** a Department of Mechanical Systems Engineering, College of Engineering, Toyama Prefectural University, 939-0398 Kurokawa 5180, Imizu, Toyama, Japan; b Laboratoire des Sciences des Procédés et des Matériaux (LSPM) - UPR CNRS 3407, Université Sorbonne Paris Nord, 99 avenue Jean-Baptiste Clément, Villetaneuse 93430, France; c Z3Dlab SAS, 2 Chemin de Coye, Chaumontel 95272, France; d Graduate School of Science and Engineering, Ritsumeikan University, 525-8577 Nojihigashi 1-1-1, Kusatsu, Shiga, Japan; e Department of Materials Physics, Eötvös Lorand University, P.O.B. 32; H-1518, Budapest; Hungary; f College of Science and Engineering, Ritsumeikan University, 525-8577 Nojihigashi 1-1-1, Kusatsu, Shiga, Japan; g Departement of Mechanical Engineering, College of Engineering, Tokyo Denki University, 120-8551 Senjuasahicho 5, Adachi, Tokyo, Japan

**Keywords:** Metal matrix composite, Rupture mechanism, Crack growth, EBSD characterization

## Abstract

The data presented here aim to show how to analyze crack propagation of a novel metallic matrix composite of Ti-6Al-4V reinforced with 1 wt.% nano-yttria-stabilized zirconia processed by laser powder bed fusion technology. The data was acquired via microstructural observations and electron backscatter diffraction (EBSD) analyses after the quasistatic tensile tests at room temperature. The overall crack path configuration based on the fracture surface observation by scanning electron microscopy (SEM) was first operated, presenting two main regions: (i) local inclined planes (hereafter denoted as “stair-like”), and (ii) region in accordance with the theoretical mode I fracture plane. Thereafter, a series of EBSD data set on a surface obtained after longitudinal cut off operation on one failed piece was conducted at three distinct positions: (i) in the stair-like configuration region, (ii) in the mode I fracture region, and (iii) in the region where the crack path made his transition between these two mechanisms. Since the EBSD data sets were not prone to any post-processing filtering operation, comparison of the observed mechanism with other Ti-6Al-4V alloy processed by additive manufacturing (AM) technology can be easily carried out.

Specifications Table

**Table:** 

Subject	Material Characterization
Specific subject area	Cracking mechanism occurring in Ti-6Al-4V alloy processed by additive manufacturing.
Data format	SEM image .tiff, OIM EBSD raw data .osc file, Microsoft Excel .xlsx file, Matlab script .m
Type of data	Table, Image, Chart, Graph, Figure
Data collection	All the data were gathered by the following devices: (i) Scanning electron microscopy (SEM) was carried out by JSM-7800F manufactured by JEOL Ltd; (ii) this same microscope was equipped with an EBSD acquisition module, which consisted of an EDAX TSL camera. The raw data were post-processed by OIM Analysis v.8.1.0 software to draw inverse pole figures (IPFs) of the Ti-α phase. Any EBSD datum which did not reach a minimum confidence index (CI) of 0.100 in accordance with the post-processing software was excluded. The Schmid factor applied on basal (<*a*>), prismatic (<*a*>), pyramidal (<*a*>), and pyramidal (<*a+c*>) slip systems (i.e., a total of 24 systems) were analyzed in some key grains.
Data source location	This series of data set were obtained at Toyama Prefectural University, Imizu Campus (City of Imizu, Toyama Prefecture, Japan).
Data accessibility	Repository name: Mendeley Data Data identification number: 10.17632/4kv67mpm7z.1 Direct URL to data: https://data.mendeley.com/datasets/4kv67mpm7z/1

## 1 Value of the Data

• The data will help characterize the mechanical behavior of nanocomposites with metal matrix (MMCs) under tension.
• The data are evidence to follow the propagation of cracks in MMCs made by laser powder bed fusion (L-PBF) technology.
• The data will help researchers in the EBSD analysis, making it possible to differentiate between the different modes of rupture of the MMCs observed on the fracture surface.
• Beyond the MMCs processed by L-PBF technology, the data analysis methodology could be applied to any AMed Ti-6Al-4V alloys with heterogeneous microstructure.
• Researchers could use these data to discuss and comprehend the cracking phenomenon in AMed Ti-6Al-4V-based MMCs.

## 2 Background

The present dataset represents experimental results from a tensile test campaign performed on Ti-6Al-4V alloy reinforced with 1 wt.% nano-yttria-stabilized zirconia processed by heat treatment at 600 °C for 2 h in an argon-protected environment and hot isostatic pressing (HIP) at 920 °C under 100 MPa for 2 h (denoted as ZTP1 HT+HIP). Indeed, the fracture surface observation has highlighted macroscopic inclined surfaces in a way similar to unreinforced AMed Ti-6Al-4V alloys. Nevertheless, as far as the authors know, no in-depth description of the related phenomenon is available in the literature at the present stage. Therefore, further investigations were conducted to clarify this irregular rupture pattern regarding the theoretical mode I plane. The present data underlines an unpreceded overview of this rupture mechanism by including SEM images and EBSD acquisitions. To achieve a complete understanding of the dataset thus obtained, full access to the original data, especially the raw EBSD inputs, was mandatory. Therefore, the authors have prepared this dataset as a companion to the original research article.

## 3 Data Description

The dataset connected to this work has the following structure. (i) The “EBSDdata” file includes the raw EBSD acquisition input (.osc files) of four distinct areas. The file name is related to the IPF map figure terminology of the present document. (ii) “Images” encloses the raw SEM images in two distinct files: the “fracture surface” file includes the fracture surface overview of eight different specimens (T1∼T8), and the “Lateral surface” file presents the observation carried out on a sliced specimen. The image name in the latter file corresponds to the figure terminology of the present document. (iii) “SF calculation,” where SF stands for Schmid factor, includes two files: “factor.m” is a Matlab Executable Script for the calculation of slip system orientation and related Schmid factor value, knowing the Ti-α grain orientation expressed by Euler angles in ZXZ rotation (Bunge convention); and a Microsoft Excel file “Allsystem.xlsx” gathering the slip system results thus obtained for several critical grains detected in EBSD analyses.

Alike unreinforced Ti-6Al-4V alloy processed by AM technology, most of the fracture surfaces of ZTP1 HT+HIP material presents one or more stair-like regions (as highlighted in our previous work [1]), where the local crack propagation does not follow the theoretical mode I fracture plane. Indeed, such a peculiar crack path propagation has already been observed on the fracture surface of unreinforced Ti-6Al-4V processed by several AM technologies, as selective laser melting (SLM) [2], electron beam manufacturing (EBM) [2,3], or laser additive manufacturing with a filler wire (LAMW) [4]. As a preliminary step to investigate this cracking mechanism, a manual assessment of the fracture surface fraction covered by this stair-like configuration on the fracture surface has been operated over height distinct specimens stressed up to rupture. The raw micrographs of the fracture surfaces are available in the repository (/Images/FractureSurface). The corresponding data have been inserted in Table 1, underlining a very scattered distribution.

**Table 1:** Stair-like configuration fraction value over the investigated specimens.

Specimen	#T1	#T2	#T3	#T4	#T5	#T6	#T7	#T8	Average	Standard deviation
Stair-like configuration fraction (%)	6.3	4.6	25.4	14.8	17.6	7.2	7.7	3.9	10.9	7.6

**Fig. 1:**
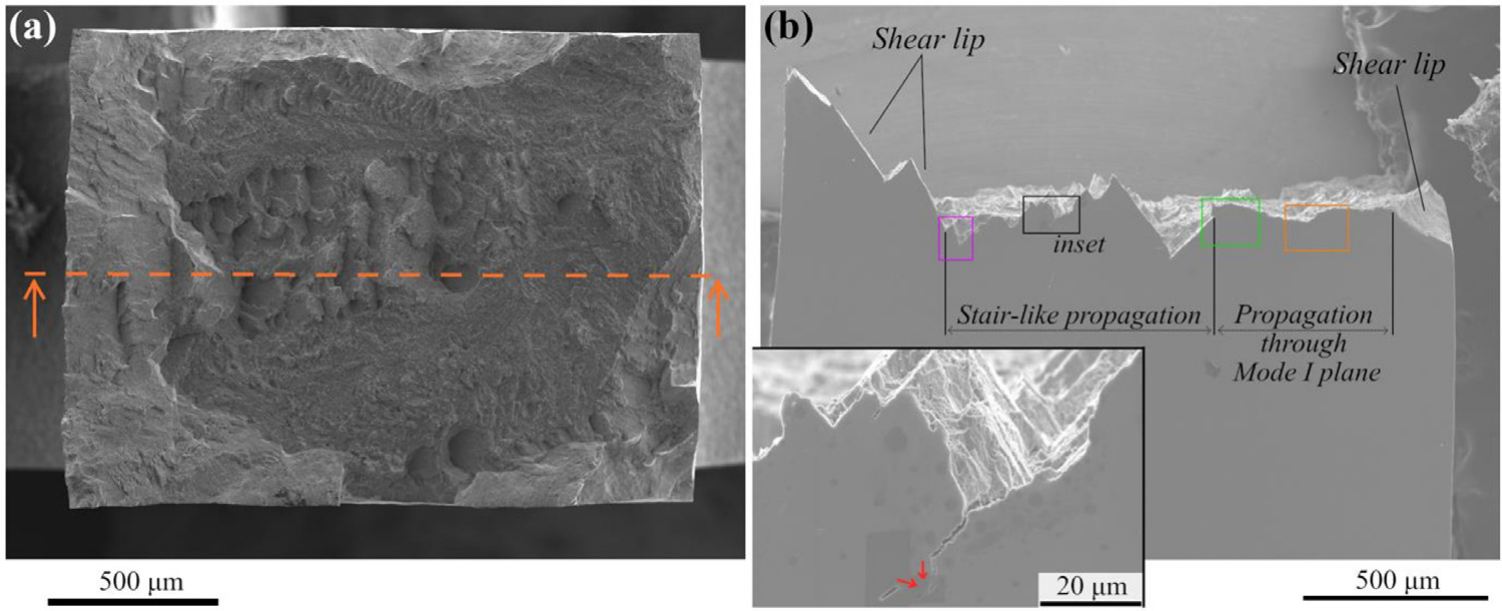
(a) Overall fracture surface of the considered fatigue specimen presenting a large stair-like crack path at its central region; and (b) surface obtained after grinding the same specimen up to the orange dashed line. The inset (black-squared region in the large image) shows a crack nucleated in a valley of the stair-like configuration, presenting a kink highlighted by red arrows.

To better comprehend this phenomenon, the failed piece depicted in Fig. 1(a) has been sliced up to the position pointed out by a horizontal orange dashed line. From this position, further EBSD investigations were carried out on the obtained lateral surface to gather decisive data on the microstructure at the vicinity of the crack. To this end, after the grinding operation, the surface was finished by colloidal silica suspension for the feasibility of EBSD experiments. An overview of the resulting surface is presented in Fig. 1(b), where regions corresponding to the three fracture aspects (i.e., stair-like configuration, Mode I propagation, and shear lip) can be easily discriminated. The raw micrographs of the lateral surfaces are available in the repository (/Images/LateralSurface).

**Fig. 2:**
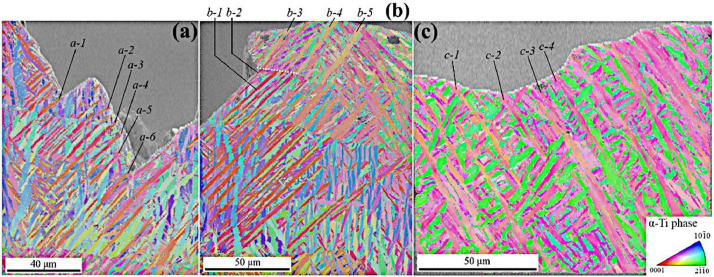
Superimposed SEM images and IPF maps obtained by EBSD with the step size of 0.35 µm. (a), (b) and (c) show the areas delineated by purple, green, and orange squares in Fig. 1(b), respectively. White dotted line in (b) underlines the *β*-prior GB where the crack path transition occurs. Loading direction is vertical for all the micrographs.

EBSD investigations were performed in three distinct zones highlighted by squares in purple, green, and orange color, and the results are shown in Fig. 2(a), (b), and (c), respectively, in the form of combined SEM images and IPF maps. The corresponding raw EBSD data are available in the form of .osc files in the repository (/EBSDdata). In accordance with the IPF map in Fig. 2(a), the crack orientation is locally driven by the primary *α*-grain family appearing in red color. Consequently, a focus on these grains is carried out via the analysis of their orientation obtained via the EBSD method. Table 2 presents the data related to the shear stress applied on each of the basal (<*a*>), prismatic (<*a*>), pyramidal <*a*>, and pyramidal <*a+c*> slips system (i.e., total of 24 systems) for primary grains *a-1* ∼ *a-6,* by the assessment of the Schmid factor values *m*. These calculations have been operated via a Matlab script available in the repository (/SFcalculation/factor.m). Furthermore, for each grain, the Schmid factor values *m* and orientation of the related slip plane on the lateral surface *θ* for any considered slip system are available in the repository (/SFcalculation/Allsystems.xlsx). In accordance with those data, every primary grain *a-1* ∼ *a-6* possesses a very significant Schmid factor among the prismatic systems, reaching a value of 0.472 on average for the six distinct grains. These results align with the data reported in [1]. Furthermore, Table 2 also reveals that the major axis of grains numbered *a*-*1* ∼ *a*-*6* presents an angle with the loading direction (hereafter referred to as *ζ* angle) close to 45°, in accordance with the post-processing method elaborated in the section dealing with data treatment method. Thus, a very high shear stress developed in these GBs during tension, contributing to easier cracking, in accordance with the discussion carried out by Liu et al. [5].

**Table 2:** Orientation and plausible activated slip system in grains indicated in Fig. 2.

Grain	Euler angles (º)			Max. shear stress prismatic plane	Schmid factor *m*	Plane trace angle *θ* (º)	*α*-grain major axis orientation *ζ* (º)
	*φ*_1_	Φ	*φ*_2_				
*a-1*	47.5	9.5	155.2	$\left(1\;0\;\bar{1}\;0\right)$	0.477	52.6	41.0
*a-2*	16.9	14.5	187.9	$\left(1\;0\;\bar{1}\;0\right)$	0.471	53.9	42.3
*a-3*	354.1	11.6	270.4	$\left(1\;\bar{1}\;0\;0\right)$	0.472	54.0	44.6
*a-4*	357.7	14.2	147	$\left(0\;1\;\bar{1}\;0\right)$	0.471	53.9	47.6
*a-5*	32.5	20.5	172.5	$\left(1\;0\;\bar{1}\;0\right)$	0.462	53.7	42.9
*a-6*	220	166.1	258.7	$\left(0\;1\;\bar{1}\;0\right)$	0.479	51.0	42.6
*b-1*	203.1	163.6	177.2	$\left(1\;\bar{1}\;0\;0\right)$	0.464	54.9	46.2
*b-2*	199.4	162.8	113.6	$\left(1\;\bar{1}\;0\;0\right)$	0.465	54.6	47.1
*b-3*	237.8	139.9	276.9	$\left(0\;1\;\bar{1}\;0\right)$	0.351	52.5	38.2
*b-4*	235.4	140.0	333.4	$\left(1\;0\;\bar{1}\;0\right)$	0.360	52.8	32.9
*b-5*	236.9	138.3	332.3	$\left(1\;0\;\bar{1}\;0\right)$	0.344	55.2	31.7
*c-1*	82.4	37.5	314.5	$\left(0\;1\;\bar{1}\;0\right)$	0.299	-59.7	-32.0
*c-2*	81.6	40.0	311.1	$\left(0\;1\;\bar{1}\;0\right)$	0.259	-64.6	-33.5
*c-3*	271.6	146.7	55.1	$\left(0\;1\;\bar{1}\;0\right)$	0.335	-58.2	-34.1
*c-4*	267.6	146.3	52.5	$\left(0\;1\;\bar{1}\;0\right)$	0.324	-59.8	-30.0

At the left side of the green square in Fig. 1(b), there is a transition from stair-like fracture surface morphology to mode I plane configuration. The corresponding EBSD image is shown in Fig. 2(b), where this sudden transition happens at a GB of a prior *β*-grain, as indicated by the white dotted line. The transition from the former prior *β*-grain to the second one induces a rotation of the primary Ti-*α* grains, which the color alteration on the IPF map can quickly figure out. The data relative to the orientations of the several primary Ti-*α* grains numbered from *b-1* to *b-5* in the considered zone were evaluated, and the results are listed in Table 2. Grains *b-1* and *b-2*, located in the prior *β*-grain before the transition, present a configuration where the Schmid factors (*m* > 0.465) and *ζ* angles are highly favorable for prismatic plastic slip activation, similar to grains *a-1*∼*a-6*. Moving to the contacting prior *β*-grain, the primary Ti-*α* grains *b-3, b-4,* and *b-5* have Schmid factors (*m* < 0.360) and *α*-lath trace angles (*ζ* < 39°) less preferential for prismatic slip system activation, resulting in more difficult early cracking phenomenon along the primary α-grain family by small crack coalescence, as reported in [1].

EBSD investigation was also performed on the region indicated by the orange square in Fig. 1(b). This zone corresponds to the location where the macroscopic crack path follows the theoretical mode I plane. The EBSD image in Fig. 2(c) reveals that the crack orientation is independent of the material microstructure. The orientation data of a few primary Ti-*α* grains (referred to as *c-1* ∼ *c-4*) have been investigated, resulting in the estimation of their Schmid factors *m* and *ζ* angles reported in Table 2. The Schmid factors of these grains for prismatic slip are even less than the one derived from grains *b-3*∼*b-5* regions (*m* < 0.335). Furthermore, the major axis of the laths has a less favorable spatial orientation for shear stress generation along GBs (|*ζ*| < 31°). This combination is detrimental for prismatic slip system activation in those primary α-grains, which hinders the early cracking phenomenon described in [1].

**Fig. 3:**
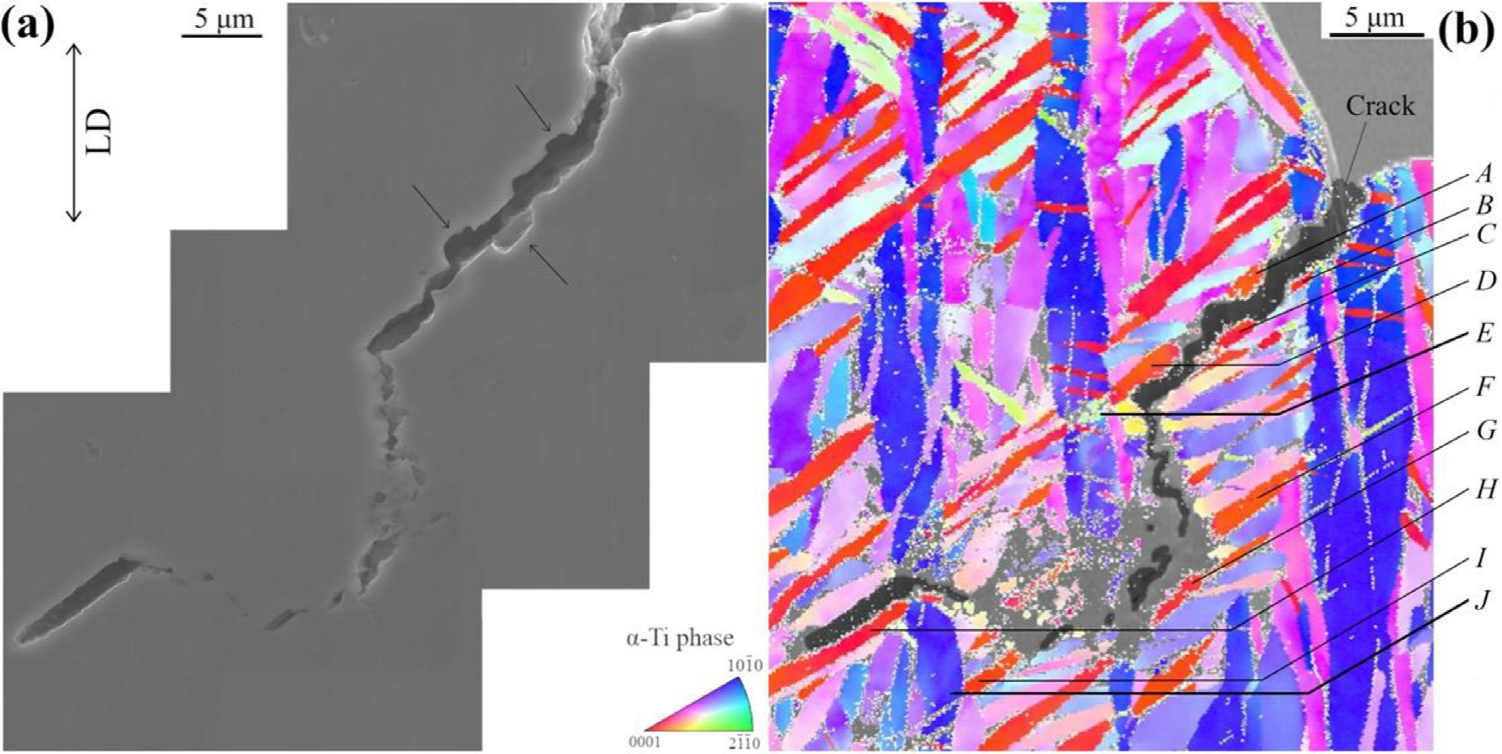
(a) High magnification SEM micrograph of a secondary crack detected in the inset of Fig. 1(b), and (b) Superimposed SEM image and IPF map of the region around the crack, where the crack is highlighted by black color. The step size is 0.15 µm. Loading direction is vertical.

Moreover, the inset of Fig. 1(b) reveals the formation of secondary cracks on the lateral surface of the tensile-tested ZTP1 samples. The crack path generates a kink, where one segment is oriented parallel to the loading direction despite this being a highly unfavored configuration for crack propagation. Therefore, an EBSD investigation was carried out on the related zone shown in the SEM micrograph in Fig. 3(a), resulting in the superimposed SEM image and IPF map displayed in Fig. 3(b). The raw image and raw EBSD data are available in the repository (/Images/LateralSurface and /EBSDdata/ScanFig3.osc, respectively). The crystallographic orientation data of the spots denoted by letters from *A* to *J* were determined and listed in Table 3. In the upper region of the analyzed zone (from spot *A* to *D*), the secondary crack presents several crenel patterns, as highlighted by black arrows in Fig. 3(a). In this region, the crack propagation path clearly follows the interface between the red primary Ti-*α* grain with high Schmid factors (see Table 3) and their neighbors, but alternates several times between the upper and the lower grain boundaries. It yields sharp deviations of the crack path in the form of crenel patterns. In addition, a very large crack can be observed along the GB of spot *H*. In accordance with the IPF map, this spot *H* consists of another primary Ti-*α* grain from the same primary grain family, thus presenting a very large Schmid factor (*m* = 0.495). However, the crack coalescence driven by the increasing tensile loading between grains {*A*∼*D*} and grain {*H*} has formed a kink pattern in-between. Data relative to Schmid factor and slip plane orientation *θ* of every considered slip system are available in the repository (SFcalculation/Allsystems.xlsx).

**Table 3:** Orientation and plausible activated slip system in locations indicated in Fig. 3(b).

Region	Euler angles (º)			Max. shear stress prismatic plane	Schmid factor *m*	Plane trace angle *θ* (º)	*α*-grain major axis orientation *ζ* (º)
	*φ*_1_	Φ	*φ*_2_				
*A*	171.6	169.1	92.8	$\left(1\;0\;\bar{1}\;0\right)$	0.495	48.3	50.4
*B*	205.1	174.3	182.9	$\left(1\;\bar{1}\;0\;0\right)$	0.484	52.1	
*C*	215.1	173.6	132.0	$\left(1\;0\;\bar{1}\;0\right)$	0.479	53.0	
*D*	217.6	168.2	198.9	$\left(1\;\bar{1}\;0\;0\right)$	0.489	48.5	
*E*	165.3	67.6	280.6	$\left(1\;0\;\bar{1}\;0\right)$	0.443	-38.7	/
*F*	49.7	10.4	150.0	$\left(1\;0\;\bar{1}\;0\right)$	0.485	49.7	56.8
*G*	50.7	6.6	211.0	$\left(1\;\bar{1}\;0\;0\right)$	0.483	51.7	
*H*	68.8	9.0	130.2	$\left(1\;0\;\bar{1}\;0\right)$	0.495	49.2	50.5
*I*	67.1	4.9	134.5	$\left(1\;0\;\bar{1}\;0\right)$	0.484	51.7	59.4
*J*	86.8	98.7	358.6	$\left(1\;0\;\bar{1}\;0\right)$	0.013	82.1	∼90

The presence of the kink is necessarily inferred by the impossibility for the crack to propagate along the primary *α*-grain {*A∼D*}. In accordance with the EBSD data, the presence of a small grain ahead of the crack path deviation, highlighted by the location *E* in Fig. 3(b), is the reason for this impossibility. Its limited size (approximately 2.5 µm length, in contrast with Ti-*α* primary grains length larger than 15 µm [1]) restricts the slip length, which is detrimental for plastic slip activation despite the relatively high Schmid factor for prismatic slip system (*m* = 0.443). The crack deviates from primary *α*-grain {*A*∼*D*} to another primary grain highlighted by spots *F* and *I* (*G* location consists of a different primary grain). In parallel, a large void observed along the primary grain *H* is promoted by the early cracking mechanism reported in [1]. In such a circumstance, a crack coalescence mechanism between the lower position of the crack along grain {*F,I*} and the upper position of grain {*H*} occurs.

## 4 Experimental Design, Materials and Methods

After EBSD acquisition completion, both the IPF maps and the original SEM image of the analyzed region have been obtained by OIM Analysis v.8.1.0 software. Original data has been filtered by the following procedure: (i) Grain CI Standardization (tolerance 5.0) and (ii) Grain Fit Standardization (tolerance 5.0). The superposition of the obtained SEM image and IPF maps has been carried out by GIMP v.2-10 image manipulation program.

Based on the Euler angles (*φ*_1_, Φ, *φ*_2_) obtained through EBSD post-processing software representing the crystallographic lattice orientation, the Schmid factors related to the 24 slip systems considered of any analyzed point owning a sufficient CI (i.e., CI > 0.100) have been computed by the Matlab executable script available in the repository (SFcalculation/factor.m). This script has been executed in Matlab R2023b.

## Limitations

Not applicable.

## Ethics Statement

The authors have read and follow the ethical requirements for publication in Data in Brief. The authors confirms that the present work does not involve human subjects, animal experiments, or any data collected from social media platforms.

## CRediT authorship contribution statement

**Benjamin Guennec:** Methodology, Formal analysis, Data curation, Software, Investigation, Visualization, Writing – original draft. **Amine Hattal:** Investigation. **Kentaro Nagano:** Investigation. **Azziz Hocini:** Validation. **Kamilla Mukhtarova:** Investigation. **Takahiro Kinoshita:** Supervision. **Noriyo Horikawa:** Supervision, Resources. **Hiroshi Fujiwara:** Supervision, Resources. **Jenő Gubicza:** Validation, Writing – review & editing. **Madjid Djemaï:** Resources. **Guy Dirras:** Conceptualization, Project administration, Writing – review & editing.

## Data Availability

Failure mechanism data related to the crack path of additively manufactured Ti-6Al-4V reiforced by nano-sized particules (Original data) (Mendeley Data).

## Appendix Supplementary Materials

Image, application 1
