# Supplementary material for: Failure mechanisms data analysis during tension of additively manufactured Ti-6Al-4V alloy reinforced with nano-zirconia particles: Investigations of the crack path
Source: Data Brief. 2024 Mar 1;54:110283. doi: 10.1016/j.dib.2024.110283 (PMC10957374; doi:10.1016/j.dib.2024.110283)
Supplement: Supplementary file 1 [file mmc1.pdf]

Materials Science & Engineering A

Strengthening and failure mechanisms during tension of a Ti-6Al-4V alloy-based nanocomposite processed by laser powder bed fusion

--Manuscript Draft--

**Manuscript Number:**	
**Article Type:**	Research Paper
**Keywords:**	Titanium alloys; Composites; additive manufacturing; Stress/Strain measurements; Electron microscopy.
**Corresponding Author:**	Benjamin GUENNEC, Ph.D.<br>Toyama Prefectural University<br>Imizu, JAPAN
**First Author:**	Benjamin GUENNEC, Ph.D.
**Order of Authors:**	Benjamin GUENNEC, Ph.D.
	Amine Hattal, PhD
	Kentaro Nagano, MSc
	Azziz Hocini, PhD
	Kamilla Mukhtarova, PhD
	Takahiro Kinoshita, PhD
	Noriyo Horikawa, PhD
	Hiroshi Fujiwara, PhD
	Jeno Gubicza, PhD
	Madjid Djemai
	Guy Dirras, PhD
**Abstract:**	The present work aims to clarify the tensile behavior of a novel metallic matrix composite of Ti-6Al-4V alloy reinforced with 1 wt.% nano-yttria-stabilized zirconia, processed by laser powder bed fusion technology. The as-processed samples were post-processed using notably hot isostatic pressing in order to investigate the most promising material for actual structural applications. The cracking behavior is governed by the activation of prismatic slip systems in primary α-Ti grains. Under horizontal loading configuration (i.e., perpendicular to building direction), average yield stress and ultimate tensile strength of 999 and 1195 MPa have been found, respectively. Strengthening mechanisms caused by (i) solid solution due to raising oxygen concentration and (ii) dispersion hardening have resulted in similar yield stress increases of 108 and 99 MPa, correspondingly. Furthermore, the effect of the texture on the mechanical properties of the investigated material was assessed via a statistical approach, which forecasts a strong strength anisotropy depending on the loading direction. In addition, a particular attention has been paid to the effect of nano-yttria-stabilized zirconia addition on strain-hardening ability, underlining that the enhanced amount of solute oxygen yielded a strong strain hardening in stage II of plastic deformation.
**Suggested Reviewers:**	Sebastien Pouzet, PhD<br>National Conservatory of Arts and Crafts<br>sebastienpouzet@hotmail.fr<br>Valuable knowledge on additive manufacturing and metal matrix composite
	Hongyu Chen, PhD<br>Ningbo University<br>chenhongyu@nbu.edu.cn<br>Valuable knowledge on metal matrix composite processing by laser-powder bed fusion technology.

Powered by Editorial Manager® and ProduXion Manager® from Aries Systems Corporation

Editor, Materials Science and Engineering A
Dear Editor,

I am pleased to submit an original research article entitled "Strengthening and failure mechanisms during tension of a Ti-6Al-4V alloy-based nanocomposite processed by laser powder bed fusion" by B. Guennec, A. Hattal, K. Nagano, A. Hocini, K. Mukhtarova, T. Kinoshita, N. Horikawa, H. Fujiwara, J. Gubicza, M. Djemai, and G. Dirras for consideration for publication in *Materials Science and Engineering A.*

Among the recent developments in the physical metallurgy field, additive manufacturing (AM) is under the spotlight of numerous societal actors, such as researchers or industrials, and consumers via the well-spread 3D printing technology. Beyond its multiple advantages (energy consumption, complex shape printing, etc.), AM also represents a new tool for more efficient fabrication processing of specific materials than conventional methods, such as high-entropy alloys or metallic matrix composites (MMCs). In line with this approach, laser-powder bed fusion melted Ti-6Al-4V blended with 1 or 2.5 wt.% of nano yttria-stabilized zirconia (nYSZ), called as materials ZTP1 and ZTP2.5, respectively, have been developed. Our research group has reported an exciting combination of mechanical properties of these materials [1-3]: high compression strength, high bulk density larger than 99.9% after hot isostatic pressing (HIP) post-treatment, and elevated high-cycle fatigue endurance.

Therefore, in a quest for improving our comprehension on the mechanical properties of such an MMC, an in-depth investigation of the quasi-static tensile behavior of the ZTP1 material has been carried out in the present work. In this manuscript, only the HIPed samples have been involved to focus our efforts on the most promising candidate for structural applications. After studying the stress/strain characteristics and the fracture surfaces features, the present work clarifies the effect of the nYSZ addition on the tensile properties of the ZTP1 material. This addition has led to the formation of yttrium-based particles, resulting in a strengthening by Orowan mechanism (increase in yield stress by approx. 99 MPa), and a similar level of solid solution hardening by rising O concentration (increase by approx. 108 MPa). Furthermore, using a statistical approach, it was revealed that the investigated material's strong $(11\bar{2}0)_\alpha$ fiber texture promotes a strong yield stress anisotropy. In the case of the horizontal loading, the extended strain hardening capacity is governed by this texture, which promotes easy cross-slip between pyramidal and basal slip systems. Such activations have been experimentally detected by electron channeling contrast imaging (ECCI) and X-ray line profile analysis (XLPA) methods. Finally, the data related to the crack propagation pattern observed in the presently investigated material is also the object of a submission to *Data in Brief*. Such documents should represent valuable resources for any researcher in the field of AMed Ti-6Al-4V alloys.

We believe that the present manuscript is appropriate for publication in *Materials Science and Engineering A.* I shall highly appreciate if you could submit the present manuscript to the reviewing procedure at your earliest convenience.

Thank you for processing our manuscript.

Sincerely,

The corresponding author, on behalf of all co-authors
Dr. Benjamin Guennec, PhD

[1] A. Hattal et al., Mater. Des. 180 (2019) 107909.
[2] A. Hattal et al., Mater. Des. 214 (2022) 110392.
[3] B. Guennec et al., Int. J. Fatigue 164 (2022) 107129.

Strengthening and failure mechanisms during tension of a Ti-6Al-4V alloy-based nanocomposite processed by laser powder bed fusion

Benjamin Guennec[a,*], Amine Hattal[b,c], Kentaro Nagano[d], Azziz Hocini[b], Kamilla Mukhtarova[e], Takahiro Kinoshita[a], Noriyo Horikawa[a], Hiroshi Fujiwara[f], Jenő Gubicza[e], Madjid Djemaï[c] and Guy Dirras[b,**]

[a] Toyama Prefectural University, College of Engineering, Department of Mechanical Systems Engineering, 939-0398 Kurokawa 5180, Imizu, Toyama, Japan.

[b] Université Sorbonne Paris Nord, Laboratoire des Sciences des Procédés et des Matériaux (LSPM) - UPR CNRS 3407, 99 avenue Jean-Baptiste Clément, 93430, Villetaneuse, France.

[c] Z3Dlab, Parc Technologique, 26 Rue des Sablons, Montmagny 95360, France.

[d] Graduate School of Science and Engineering, Ritsumeikan University, 525-8577 Nojihigashi 1-1-1, Kusatsu, Shiga, Japan.

[e] Department of Materials Physics, Eötvös Lorand University; Budapest; P.O.B. 32; H-1518, Hungary.

[f] College of Science and Engineering, Ritsumeikan University, 525-8577 Nojihigashi 1-1-1, Kusatsu, Shiga, Japan.

* Corresponding author. e-mail: bguennecpro@gmail.com, ** e-mail: guy.dirras@lspm.cnrs.fr

Abstract

The present work aims to clarify the tensile behavior of a novel metallic matrix composite of Ti-6Al-4V alloy reinforced with 1 wt.% nano-yttria-stabilized zirconia, processed by laser powder bed fusion technology. The as-processed samples were post-processed using notably hot isostatic pressing in order to investigate the most promising material for actual structural applications. The cracking behavior is governed by the activation of prismatic slip systems in primary α-Ti grains. Under horizontal loading configuration (i.e., perpendicular to building direction), average yield stress and ultimate tensile strength of 999 and 1195 MPa have been found, respectively. Strengthening mechanisms caused by (i) solid solution due to raising oxygen concentration and (ii) dispersion hardening have resulted in similar yield stress increases of 108 and 99 MPa, correspondingly. Furthermore, the effect of the texture on the mechanical properties of the investigated material was assessed via a statistical approach, which forecasts a strong strength anisotropy depending on the loading direction. In addition, a particular attention has been paid to the effect of nano-yttria-stabilized zirconia addition on strain-hardening ability, underlining that the enhanced amount of solute oxygen yielded a strong strain hardening in stage II of plastic deformation.

Keywords: Titanium alloys; Composites; Additive manufacturing; Stress/Strain measurements; Electron microscopy.

1. Introduction

Among recent advancements in metallurgy, additive manufacturing (AM) is one of the most promising, especially considering its possible application in numerous

industrial fields. This technology presents many advantages induced by its specific layer-by-layer process fabrication, such as less offcut, complex shape parts fabrication feasibility, or its low energy consumption for part fabrication [1–5]. Furthermore, AM provides a way more practical path to creating metallic matrix composites (MMCs) than the conventional methods, such as casting technique [6] or powder metallurgy [7]. Several attempts for AM-processing of ceramic or oxide-reinforced metallic materials have already been made, using B_4C, TiC, TiB [8–11], SiC [12], reduced graphene oxide [13] or Cr_3C_2 [14] as strengthening particles. These studies reported an increased mechanical strength due to these additions. Beyond the effect the dispersion strengthening induced by dislocation pinning mechanism well assessed by Orowam model [11,12], Ti-based MMCs have exhibited a thinner α-layer microstructures [8,10,11,13,14]. It contributes to the enhanced yield stress in accordance with the Hall-Petch law. This microstructure alteration is undoubtedly related to the influence of the strengthening particles on the AM processing [8,11,12,14].

In line with its high-hardening potential, good thermodynamical affinity toward Ti-alloys [15], and high fracture toughness [16], zirconia represents a promising candidate as a strengthening particle in Ti-based materials. For this reason, a few attempts have already been made to develop zirconia-reinforced Ti-6Al-4V materials processed by AM technology. Choi et al. [17] have reported the effect of yttria-stabilized zirconia on the mechanical strength of a Ti-6Al-4V matrix consolidated by direct energy deposition (DED) process. Since the peak temperature induced by the laser beam is probably higher than the melting point of the nano-yttria-stabilized zirconia (nYSZ), solute Zr and O elements can diffuse into both α- and β-Ti phases [18,19]. Contrary, the low solubility of yttrium (<< 1 at. % and < 2 at. % in α and β phases, respectively [20]) promotes the segregation of this element, resulting in the precipitation of Y_2O_3 particles

in the cooling step of DED processing. Recently, a Ti-6Al-4V alloy matrix reinforced with 1.0 or 2.5 wt.% nYSZ were processed by laser powder bed fusion (L-PBF) technique, and the obtained samples are denoted as ZTP1 and ZTP2.5, respectively. In former reports, our research group has already depicted some fundamental aspects related to such nYSZ-reinforced Ti-6Al-4V nanocomposites [21–24]. Along with the substantial improvement of the compressive strength, the addition of strengthening particles tends to alter the microstructure, inducing grain refinement in AM-processed materials, distinctly decreasing the α-Ti lamellar length [21,22]. ZTP1 and ZTP2.5 materials have been subjected to AMS 2801 standard hot isostatic pressing (HIP) post-treatment, resulting in an increasing trend of bulk density compared with their non-HIPed counterparts [23]. This phenomenon reflects the curation of the structural defects inherent to AM processing (i.e., lack of fusion and gas-induced pores), which undoubtedly enhances the compressive strain at rupture [23]. Furthermore, reinforcement in ZTP1 subjected to HIP has also induced a noticeable change in the texture of the obtained material, leading to a strong $(0002)_\alpha$ component and an intense $(11\bar{2}0)_\alpha$ fiber along the building direction (BD) [23]. In a recent report dedicated to the study of the high-cycle fatigue properties of the ZTP1 material [24], the HIP post-processing has suppressed the occurrence of fatigue crack initiation induced by lack-of-fusion defects, which led to a considerable improvement of the fatigue endurance by a ratio of two in comparison with its non-HIPed counterpart. Consequently, such nYSZ-reinforced Ti-6Al-4V MMCs seem a promising solution for the direct applicability of AMed Ti-alloys for structural applications.

It is noted, however, that comprehending the deformation mechanisms related to the mechanical properties of such nanocomposites still requires additional researches. In-depth characterization of the tensile properties and the related strain-hardening

mechanism of nYSZ-reinforced Ti-6Al-4V MMCs needs to be performed, especially regarding the effect of the strengthening particle addition. Therefore, in this study a comprehensive investigation of the quasi-static tensile behavior of HIPed ZTP1 material is carried out. In addition to a detailed analysis of the damage mechanism via thorough observations of ruptured specimens, the strain-hardening behavior is studied and the underlying mechanisms are revealed.

2. Experimental procedure

2.1. Manufacturing processes of the investigated material

Ti-6Al-4V reinforced with 1.0 wt.% nYSZ (i.e., ZTP1) was fabricated by mixing Ti-6Al-4V and nYSZ powders and then melted by the L-PBF technique. The initial powder made of Ti-6Al-4V alloy, possessing a chemical composition given in Table 1, has an average particle size of 20 μm. The nYSZ powder is a 3 mol% yttria-stabilized tetragonal zirconia with an average particle size of 40 nm and a chemical composition tabulated in Table 2. The samples were manufactured by an SLM125 HL machine from SLM Solution GmbH, using parameters identical to our previous study [21]. Further details about the powder blending protocol and L-PBF processing can be found in [22].

Table 1 Chemical composition of the initial Ti-6Al-4V powder

Element	Ti	Al	V	O	C	N	H	Fe
wt. %	*Bal.*	6.2	4.0	0.07	0.01	0.02	0.002	0.14

Table 2 Chemical composition of the initial nYSZ nanopowder (Data from USNano™)

Particle	ZrO_2	Y_2O_3	Al	Mg	Si	Ca	S	Nb
ppm	*Bal.*	51000	20	65	102	75	165	119

Our previous work [24] dealing with the fatigue performance of the L-PBF-processed ZTP1 material showed that HIP post-treatment has resulted in a clear improvement in the fatigue strength, such that the fatigue endurance of ZTP1 is at least equivalent to Ti-6Al-4V obtained by conventional wrought processing. This outcome underlines that reinforcement by nano-yttria-stabilized zirconia is not detrimental to the durability of the whole composite in terms of fatigue properties, which suggest that such a material can be a valuable candidate for structural applications. Under such a circumstance, a thorough analysis of the damage and strengthening mechanisms under tensile loading of this material should of great interest to the scientific community. Consequently, in a way similar to [24], after L-PBF processing all specimens were subjected to a two-step post-treatment as follows: (i) a stress relief heat treatment carried out at 600 °C for 2 h in an argon-protected environment (AMS 2801 standard); and (ii) a F3001 standard HIP treatment for Ti-6Al-4V material (920 °C, 100 MPa, 2 h), afterward. For the sake of continuity with our previous works [21–24], this material will be referred to as “HT+HIP” hereafter.

2.2. Tensile testing procedure

After post-processing, specimens used for the tensile testing procedure were cut out from the as-processed plates by an electrical discharge machining (EDM) device. These specimens have a dog-bone shape with the dimensions shown in Fig. 1. The nominal width and thickness at the specimen gauge are w = 2 and t = 1.5 mm, respectively. After machining, grinding using SiC papers has been applied to remove the volume affected by the cutting process. In the final step of this process, #2400 grinding paper was used. Some surfaces were additionally polished with silica

suspension for microstructure study performed by electron backscatter diffraction (EBSD) technique. It should be noted that BD of the L-PBF process is parallel to the transversal direction (TD) of the samples, as defined in Fig. 1. Therefore, the loading direction (LD) is orthogonal to the building one, similar to the fatigue test performed in our previous work [15].

The tensile tests were carried out at room temperature in air. The dimensions of the specimens follow the JIS Z2241 standard, except the gauge length (GL) of 5 mm, which is half of the recommended value, due to limited volume of the available material. The strain was recorded by a 2-strain gauge system (strain gauge length of 1 mm) attached to the LD-TD surface in the middle of the specimens. All the tests were performed at a constant cross-head velocity of 0.1 mm/min, corresponding to an initial strain rate of 3.33×10^{-4} s^{-1}. The reduction of area was calculated from the fracture surface observation by SEM.

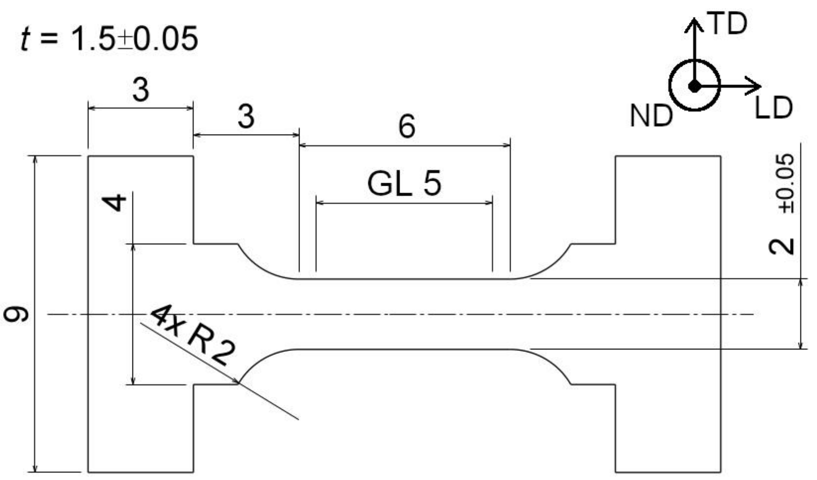

Fig. 1 Specimen shape and dimensions in mm for tensile test.

2.3. Characterization of the microstructure

Scanning electron microscopy (SEM) using the FEG SUPRA 40VP-ZEISS microscope was used to characterize the initial microstructure. SEM-EBSD investigation with the scan step size of 80 nm was carried out on an electropolished surface area of 110 μm × 110 μm. OIM Analysis v.7 software was used to perform EBSD data analysis. The microstructure of the specimens after tensile test was studied by a JEOL JSM-7800F SEM device operating at an acceleration voltage of 15 kV. The same device equipped with an EDAX TSL camera was used for the EBSD investigation of the microstructure on the LD-TD surface of some specimens. The EBSD images were post-processed by OIM Analysis v.8 software. Energy dispersive X-ray spectroscopy (EDS) was involved for the chemical analysis of the samples using the same microscope equipped with a JEOL EX-37270VUP sensor. Density measurements were performed using an Accupyc II 1340 He pycnometer on samples cleaned in an ultrasonic bath and dried for 24 hours. Further details are given in [25]. The relative density of the ZTP1 was obtained from the measured and the theoretical densities where the latter one was calculated from the nominal densities of Ti-6Al-4V and nYSZ using the rule of mixture.

Moreover, X-ray line profile analysis (XLPA) was performed to assess the crystallite size and the dislocation density [26]. The X-ray diffraction (XRD) patterns were then measured by an RA-MultiMax9 type high-resolution rotating anode diffractometer (Rigaku Corporation), using CuKα_1 radiation at a wavelength of λ = 0.15406 nm. The measured peak profiles of the Ti alloy matrix were evaluated by the convolutional multiple whole profile (CMWP) fitting procedure [27]. In this method, the diffraction pattern is fitted by the sum of a background spline and the diffraction peaks obtained as the convolution of the measured instrumental peak and the theoretical profiles caused by the finite crystallite size and the dislocations. The

instrumental peaks were measured on a standard NIST SRM660a LaB_6 sample. Under these circumstances, the area-weighted mean crystallite size and the dislocation density were determined by the CMWP procedure. In addition, the population of the different dislocation slip systems can also be obtained by analyzing the dislocation contrast factors determined by the CMWP method. An overview of one of these analyses has been depicted in Fig. S1 of the Supplementary material, where an excellent agreement between the measured and the fitted patterns can be observed. More details of this evaluation procedure are given in [26]. Furthermore, the fractions of the remanent Ti β-phase were estimated by the intensity fraction of this phase in the whole diffractogram. Such analyses were carried out on a surface etched with HF for 20 s to avoid any influence of the specimen surface grinding and polishing on the results.

Complementary investigations by electron channeling contrast imaging (ECCI) of the ruptured specimens were also carried out with a JEOL JSM-7200F SEM. To this end, the specimen was electropolished to suppress the influence of grinding operation. Finally, the identification of slip planes related to plastic features was performed as follows. Considering basal (<*a*>), prismatic (<*a*>), pyramidal <*a*>, and <*a*+*c*> slip systems (i.e., a total of 24 systems), the orientation of the slip plane trace on the sample surface (denoted as θ) was compared with the theoretical ones computed via the crystallographic orientation obtained by the EBSD analysis. Irrespective to LD direction, the horizontal axis of the SEM image is the reference direction for the θ angle definition. In the present paper, the orientation of the crystals will be defined by their Euler angles (φ_1, Φ, φ_2), expressed in Bunge notation (rotation ZXZ).

3. Experimental results and discussion

3.1. Microstructure of the ZTP1 HT+HIP composite material and a comparison with its unreinforced counterpart

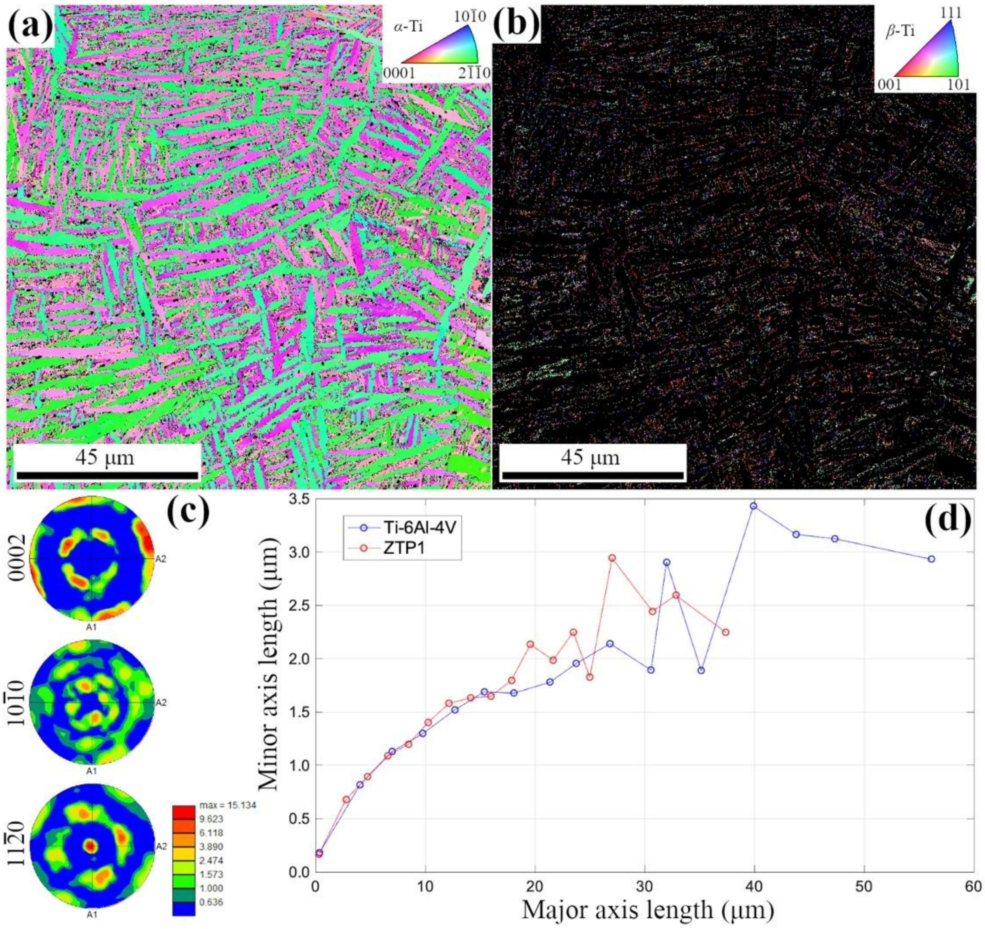

Fig. 2 Microstructure of a ZTP1 HT+HIP material. IPF maps with HAGBs indicated by black lines taken on a surface orthogonal to BD (a) for α-Ti and (b) β-Ti phases; (c) pole figures for α-Ti phase as obtained from the EBSD image in (a), where A3 corresponds to BD; and (d) the thickness versus the length of α-Ti grains shown in (a). The EBSD scan step size is 80 nm.

The ZTP1 HT+HIP material has a very high level of relative density of 99.99 ± 0.01 %. XRD revealed that the main phase is hexagonal close-packed (HCP) α-Ti with a secondary BCC β-Ti phase having the composition of $Ti_{0.7}V_{0.3}$ (card No. 01-081-9817).

The XRD peak intensity fraction of the Ti β-phase is 4 ± 1 % which is in a reasonable agreement with the area fraction of this phase as obtained from the EBSD image shown in Fig. 2 (5.1 %). It should be noted that the manufacturing process of the samples in this study was exactly the same as in our previous work investigating the fatigue properties of the ZTP1 HT+HIP material [24]. The microstructure is shown in the inverse pole figure (IPF) maps obtained by EBSD and presented in Fig. 2(a) and (b) for α and β-Ti phases, respectively. A former study suggested that the thermal history of the L-PBF process induced a preferred crystallographic orientation of α-Ti grains [28]. Indeed, the pole figures of the α-phase in Fig. 2(c) reveal a strong $(11\bar{2}0)_\alpha$ fiber texture around the BD axis (A3 direction of pole figures), in accordance with our previous work [23]. This feature is thought to influence the mechanical properties of the investigated material. Further discussion on this point will be presented in Section 4.

The morphology of the α-Ti grains was also evaluated from the EBSD IPF map using elliptical grain shape fitting. Thus, the length and the thickness of each grain were determined as the dimensions of the major and minor axes of the fitted ellipse, respectively. The α-Ti grain thickness (minor axis length) versus its length (dimension of major axis) is plotted in Fig. 2(d). For grain length smaller than 15 µm in ZTP1 HT+HIP material, an increase in α-Ti grain length is accompanied to a relatively stable increase in grain thickness. On the other hand, above the grain length of ~15 µm, the sharper fluctuations can be caused by the relatively low number of grains in the bins related to long-length grains. For comparison, the same evaluation was carried out for the unreinforced Ti-6Al-4V alloy sample in HT+HIP condition on the basis of the EBSD study reported in [23]. Although, the trend is similar for both Ti-6Al-4V and ZTP1 samples, one can notice that the unreinforced Ti-6Al-4V alloy contains larger α-Ti grains than its ZTP1 counterpart. On the other hand, the dependence of the aspect

ratio on the grain size was not influenced by the addition of the nYSZ particles. It was assumed that the primary α-Ti grains thickness is not governed by their length; therefore, length of primary α-Ti grains corresponds to the thickness fluctuation region in Fig. 2(d). Accordingly, the primary α-grains are defined as the grain having a length larger than 15 μm. Based on this definition, the average thickness of the primary α-Ti grains based on actual acquisition data is 1.9 μm for both ZTP1 and unreinforced Ti-6Al-4V materials in HT+HIP condition. In other words, the primary α-grain thickness is not influenced considerably by the addition of 1.0 wt.% nYSZ. Moreover, the secondary α-Ti grains are defined as the grains having a length at least one-third of the minimum length of the primary grains, i.e., a minimum length of 5 μm.

3.2. Stress-strain behavior of ZTP1 HT+HIP material

An example of the stress-strain behavior of the ZTP1 HT+HIP material is depicted in Fig. 3, showing the engineering and true stress-strain curves up to the beginning of the necking phenomenon. Both curves reveal a remarkable strain-hardening during tension. Based on seven distinct tensile specimens, a summary of the tensile properties of ZTP1 HT+HIP material is given in Table 3. Furthermore, Table 4 summarizes experimental tensile test results reported in former studies for unreinforced Ti-6Al-4V alloys manufactured by AM technologies and post-processed using the following treatments: (i) annealing, (ii) solution treating (ST) and (iii) HIP [29–35]. Since the orientation of LD may affect significantly the tensile properties of AMed Ti alloys [32], Table 4 also indicates the orientation of LD in respect with BD (i.e., horizontal or vertical loading).

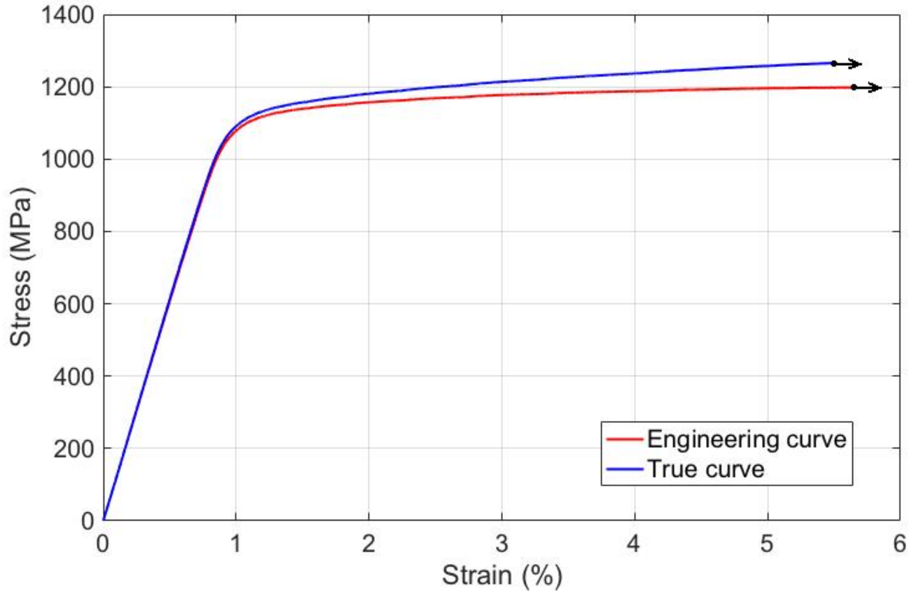

Fig. 3. Tensile stress-strain curves (both engineering and true curves) for material ZTP1 HT+HIP, where arrows represent the start of the necking phenomenon.

Table 3. Mechanical properties of ZTP1 composite in HT+HIP condition.

Material	Elastic modulus, *E* (GPa)	Yield stress 0.2%, σ_y (MPa)	Ultimate tensile strength, σ_{UTS} (MPa)	Elongation to failure (%)	Area reduction (%)
ZTP1 HT+HIP	119	999±45	1195±10	18.7±1.5	24.1±4.0

Table 4. Comparison of the quasi-static tensile properties of ZTP1 composite with those of unreinforced Ti-6Al-4V samples processed by AM [29–35].

Material	AM processing*	Post-processing*2	Loading direction orientation*3	Yield stress, σ_y (MPa)	Ultimate Tensile strength, σ_{UTS} (MPa)	Relative hardening parameter, h (%)	Elongation to failure ε_{max} (%)	Ref.
ZTP1	SLM	HT+HIP	horizontal	999±45	1195±10	19.6	18.7	Present study
Ti-6Al-4V	DMLS	Annealing	horizontal	1086	1165	7.3	15	[29]
Ti-6Al-4V	EBM	Annealing	horizontal	868	972	12.0	15	[29]
Ti-6Al-4V	SLM	Annealing	vertical	1076	1189	10.5	14	[30]
Ti-6Al-4V	SLM	Annealing	horizontal	936	1048	12.0	18.8	[31]
Ti-6Al-4V	SLM	Annealing	vertical	904	955	5.6	13.6	[31]
Ti-6Al-4V	SLM	STA	vertical	998	1063	6.5	10.6	[32]
Ti-6Al-4V	SLM	STA	horizontal	917	1024	10.7	12.4	[32]
Ti-6Al-4V	SLM	ST	vertical	919	1084	18.0	14.7	[33]
Ti-6Al-4V	SLM	STA	vertical	1086	1189	9.5	14.7	[33]
Ti-6Al-4V	DMLS	HIP	horizontal	894	997	11.5	18	[29]
Ti-6Al-4V	EBM	HIP	horizontal	774	896	15.8	18	[29]
Ti-6Al-4V	SLM	HIP	vertical	907	1022	12.7	18	[30]
Ti-6Al-4V	SLM	HIP	vertical	912	1005	10.2	8.3	[34]
Ti-6Al-4V	SLM	HIP	vertical	885	973	9.9	19.0	[35]

*SLM: selective laser melting, DMLS: direct metal laser sintering, EBM: electron beam melting; *2ST: Solution treated, STA: Solution treated and aged; *3Horizontal: LD ⊥ BD, Vertical: LD // BD.

Solution or annealing treatment is used to relax internal stresses in as-build Ti-6Al-4V materials which yielded only a very low alteration of the initial microstructure [32,33]. On the other hand, HIP post-treatment can fill the flaws inherent to SLM processed alloys, but it also causes grain coarsening. As a result, solution treatment is usually more effective than HIP from the tensile strength viewpoint. This idea is supported by the data related to unreinforced Ti-6Al-4V materials in Table 4, since the yield stress and ultimate tensile strength values for the HIPed samples are significantly lower than for their STed and annealed counterparts. Contrariwise, ZTP1 HT+HIP material reached an ultimate tensile strength comparable to STed and annealed Ti-6Al-4V counterparts. This observation can be explained by the compensation of the softening caused by the grain corsening due to HIP by the strengthening effect of the

nYSZ particles, which was also detected under compression loading in a previous report [21].

Focusing on ZTP1 HT+HIP tensile performance, one can notice a significant difference between the yield stress and ultimate tensile strength values, which is a consequence of strain hardening that is also visible in the true stress-strain curve in Fig. 3. For the sake of a straightforward comparison of the hardening capacity of the different materials, a hardening parameter calculated as $h = (\sigma_{UTS} - \sigma_{y})/\sigma_{y}$ is also reported for each material in Table 4. ZTP1 HT+HIP has the highest hardening parameter h = 19.6% among the listed materials. This aspect will be further discussed in Section 4.2. Finally, a relatively evelated elongation to failure of 18.7% is obtained. It should be noted, however, that this value is also influenced by GL. Since the present experiment considers a GL smaller than the standard one, the test procedure may result in an overestimation of the elongation at failure. Nevertheless, adding 1.0 % nYSZ particles to Ti-6Al-4V alloy did not induce a substantial decrease in overall ductility compared to its unreinforced counterparts.

3.3. Fracture surface investigation

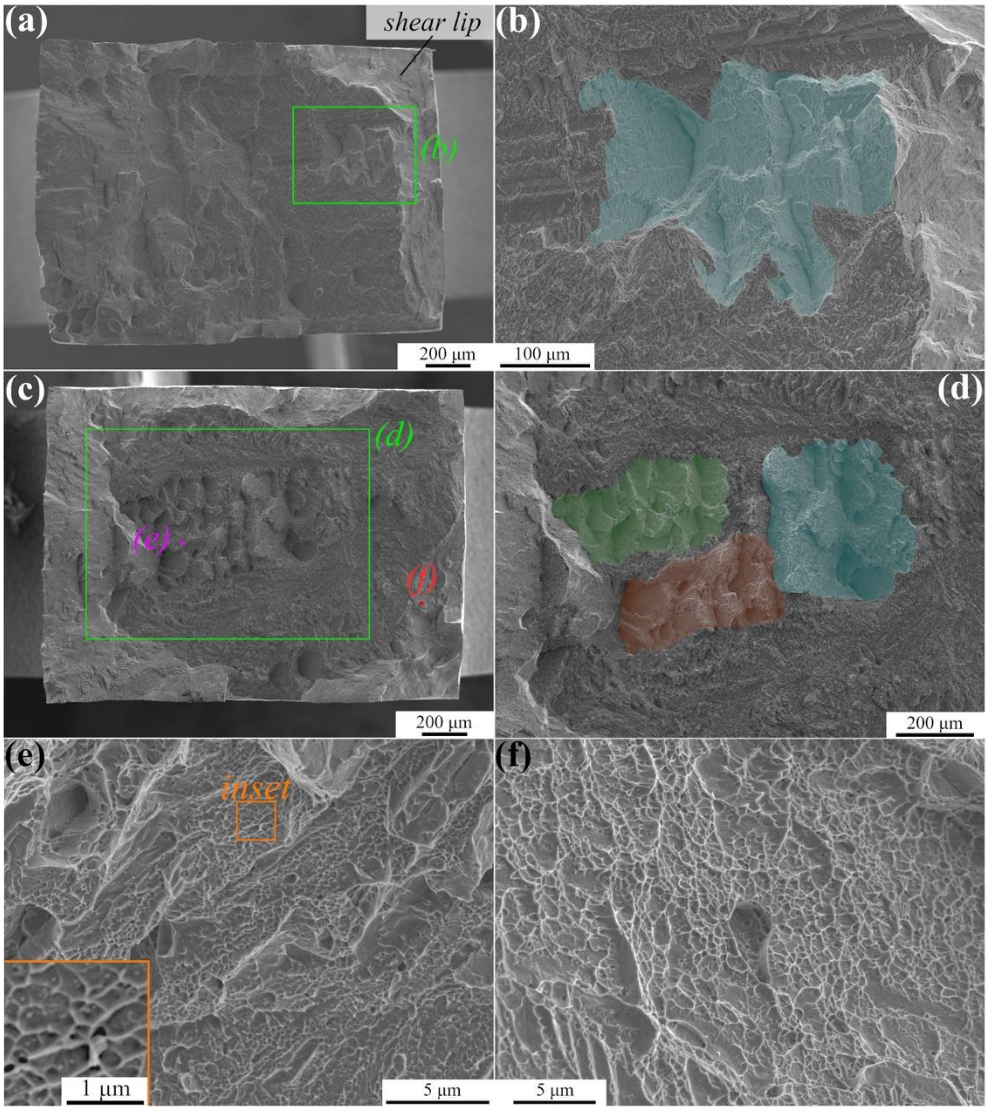

Fig. 4 SEM images taken on the fracture surface for material ZTP1 HT+HIP. (a) Overall fracture surface for a specimen having σ_{UTS} = 1200 MPa; (b) Magnified image of the region indicated by the green square in (a); (c) Overall fracture surface of another specimen having σ_{UTS} = 1160 MPa; (d) Magnified image of the region highlighted by the green square in (c); (e) High magnification micrograph taken at the location indicated by a purple point in (c), where the inset reveals the presence of nanosized particles in the center of dimples; and (f) High magnification micrograph at the location marked by the red point in (c).

Specimen fracture surfaces were investigated by SEM, resulting in the typical micrographs shown in Fig. 4. An overview of the fracture surface of a sample is presented in Fig. 4(a), which reveals three main fracture aspects. Paying attention to the green-squared area in Fig. 4(a), represented in Fig. 4(b), a region highlighted by a cyan overlay is characterized by a series of inclined planes forming a stair-like surface profile. Such a pattern has already been reported for unreinforced Ti-6Al-4V alloys manufactured by AM technology [36–38]. A thorough analysis of the stair-like configuration pattern has been undertaken in a *Data in Brief* companion document [39]. Outside this stair-like region, the fracture surface aspect consists of a macroscopically flat configuration perpendicular to LD, in line with the mode I fracture plane. Moreover, every fracture surface has generated inclined planes in the vicinity of the specimen lateral surfaces, which are related to the phenomenon of shear lips.

The fracture surface images obtained from another specimen has been inserted in Fig. 4(c-f). The overall fracture surface configuration is given in Fig. 4(c), where the stair-like configuration area seems to be divided into three different locations, indicated by orange, green, and cyan overlays in Fig. 4(d). Typical fracture surface aspects obtained at larger magnification are depicted in Fig. 4(e) and (f) (purple and red points indicate the corresponding locations in Fig. 4(c), respectively), where dimple patterns have been detected. Consequently, the fracture of such nYSZ-reinforced Ti-alloy is essentially driven by a ductile mechanism in a way similar to its unreinforced counterparts [38,40,41]. Nevertheless, in such unreinforced materials, the size of the dimples is in the order of the α-grain lamellar thickness [37,38]. As underlined in Section 2.1, even though the investigated material possesses an average primary α-grain layer thickness of about 1.9 μm, the dimple dimensions are clearly in the submicron range. This trend has already been reported in ceramic-reinforced metallic materials

[41]. Furthermore, as represented in the inset of Fig. 4(e), observation carried out at very large magnification reveals the presence of several nanosized particles at the centers of these dimples.

The fracture surface was additionally studied by EDS method. In this analysis, SEM micrographs with large magnifications (from 60,000 to 90,000) were taken at several locations on the fracture surface shown in Fig. 5(a). One example of this procedure is depicted in Fig. 5(b), where approximately 20 nm-diameter white particles are discernible inside the cavity of almost every dimple. As indicated in Fig. 5(b), EDS analysis has been conducted on nine particles (numbered from *1* to *9*). The chemical analysis results corresponding to these locations are listed in Table 4, which also shows the chemical composition of the whole surface depicted in Fig. 5(b). Due to the restricted level of spatial resolution of the SEM-EDS technique at such magnifications, this procedure cannot quantify each chemical element accurately in the nanosized particles. Indeed, this is illustrated by the high level of elements constitutive of the material matrix (i.e., Ti, Al, and V) in each position. Nevertheless, every location exhibits a high Y element concentration (from 1.18 to 3.41 wt.%) compared to the value measured on the whole area (0.04 wt.%). On the other hand, the concentration of Zr element in the scrutinized locations fluctuates from 0.11 to 1.26 wt.%, and the Zr content measured on the whole surface area (0.6 wt.%) corresponds to their average. These results are in line with the recent report by Choi et al. [17], where blending of Ti-6Al-4V alloy and nYSZ powders and subsequent consolidation by DED technology has led to the formation of yttria (Y_2O_3) strengthening particles without traces of zirconium. Due to its high solubility in both α and β titanium phases [18,19], the zirconium element is highly suspected to diffuse into the matrix by the extreme temperature rise induced by the laser-based high energy density AM process. Contrariwise, the yttrium element is

detected at very high levels at the locations corresponding to the strengthening particles. Since the SEM-EDS represents a semi-quantitative technique at such elevated magnifications, quantifying the exact chemical compositions of these strengthening particles requires further experimental efforts. Although the particles in the SEM images may be yttria (as reported in [17,38]), the formation of complex oxides as Y-Ti-O or Y-Al-O [42] cannot be excluded at the present stage.

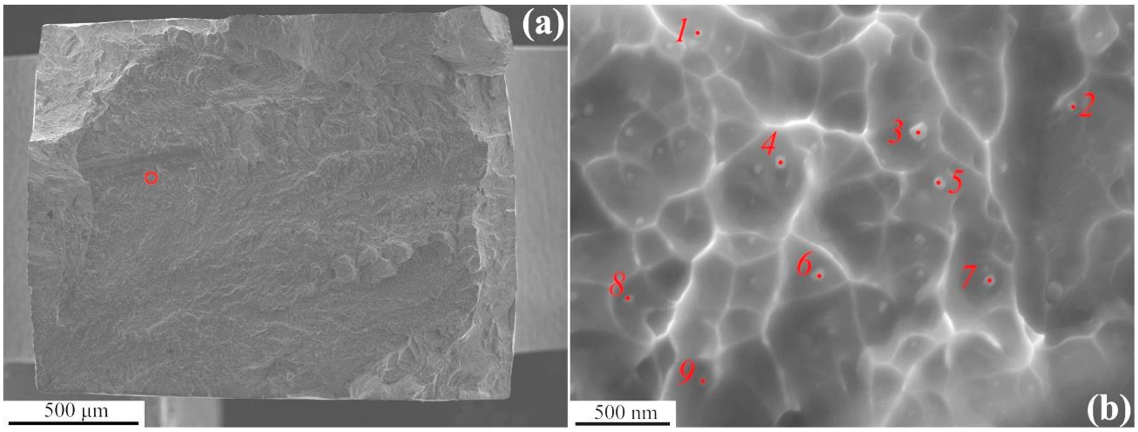

Fig. 5. (a) SEM micrograph taken on the fracture surface of a ZTP1 specimen with σ_{UTS} = 1190 MPa. The red circle indicates the area where EDS analyses was performed; (b) High-magnification micrograph of the zone selected for EDS analysis, where the positions of the local EDS measurements are numerated from *1* to *9*.

Table 4. Chemical composition of the selected zone and at positions highlighted in Fig. 5(b) obtained through EDS analysis (wt.%).

Element	*Whole area*	*1*	*2*	*3*	*4*	*5*	*6*	*7*	*8*	*9*
O	-	0.6	2.7	4.0	2.2	4.0	2.8	2.2	0.6	-
Al	6.1	5.6	6.8	6.5	5.6	6.6	4.5	6.2	5.7	4.4
Ti	82.8	77.9	80.2	78.6	77.1	79.3	73.6	80.2	84.7	79.0
V	7.5	9.6	5.9	4.3	10.6	5.4	12.0	4.3	5.1	10.1
Fe	0.5	1.5	0.8	-	0.3	0.5	1.8	-	-	0.8
Y	0.04	1.6	1.3	3.4	1.7	1.4	3.0	2.6	1.2	2.0
Zr	0.7	0.4	-	0.8	0.1	0.6	0.5	1.3	0.2	0.2

Since the secondary phase particles seem to be located roughly at the center parts of the dimples, it can be assumed that local cracks are originally nucleated in the direct vicinity of these particles. Then, these local cracks tend to propagate simultaneously to form the typical "cup and cone" geometry. In addition, no anomalous variations of the dimple size were observed. Considering that the distance between particle positions controls the dimple size, it suggests that there is a high degree of uniformity in the spatial distribution of particles within the matrix. In line with the discussion carried out by Choi et al. [17], this result should be caused which by the extensive local temperature gradient during AM processing activating Marangoni convection phenomenon [17,43,44].

3.4. Early crack damage analysis

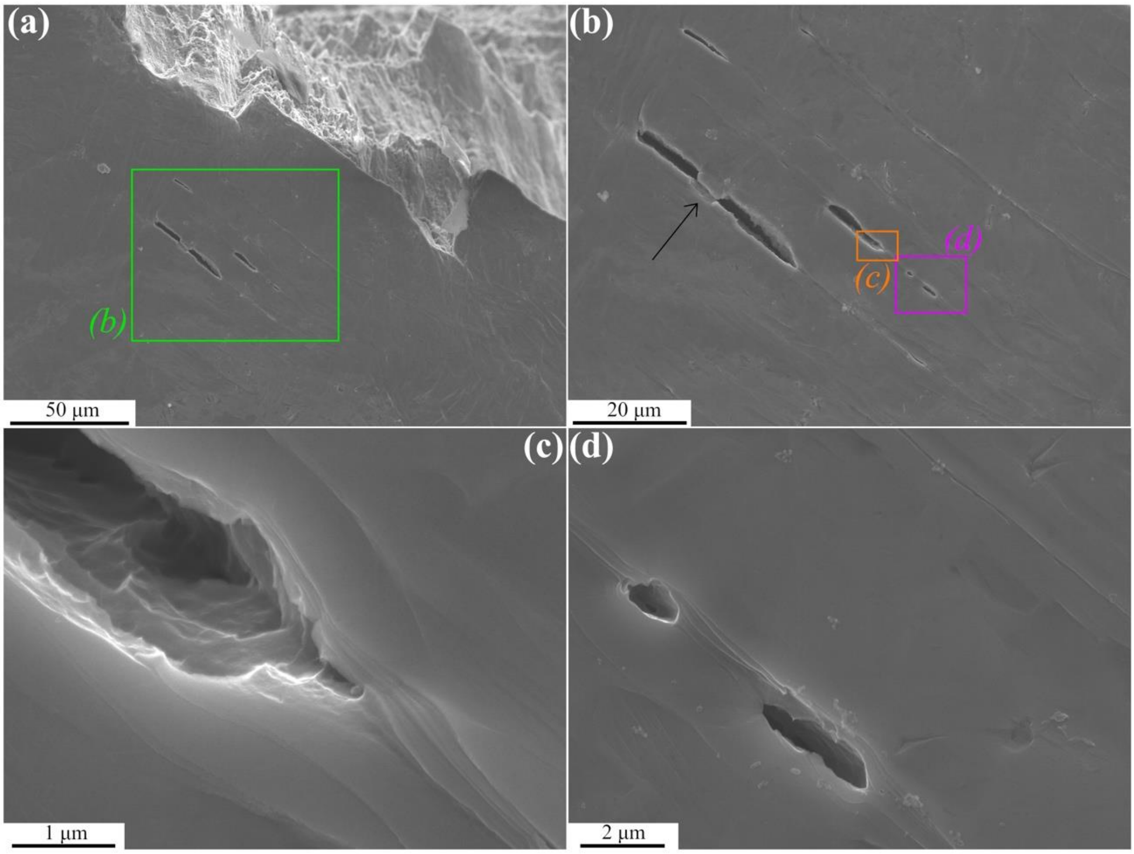

Fig. 6 SEM observation of the lateral surface (plane TD-LD) of a failed specimen with σ_{UTS} = 1190 MPa. (a) Observation of secondary cracks through a series of parallel slip bands; (b) Observation of these early cracks at larger magnification in the area indicated by the green square in (a); (c) High magnification micrograph focused on the crack tip in the region marked by the orange square in (b); and (d) small cracks generated along the same slip band concentrated region at the location indicated by the purple square region in (b).

SEM study of secondary cracks on the lateral surface of a tensile specimen was also conducted to scrutinize the crack nucleation mechanism. Typical micrographs obtained from one failed specimen are shown in Fig. 6. As revealed by Fig. 6(a), some early cracking features can be observed near the fracture surface, discernable at the upper region of the photograph. The green-squared region in Fig. 6(a) was enlarged in Fig. 6(b), showing a series of parallel plastic features which are most probably responsible for crack nucleation phenomenon. In particular, the black arrow indicates that the crack path has been subjected to a slight path irregularity of approximately 1.9 μm. Furthermore, micrographs taken with larger magnifications in the regions defined by the orange and purple squares are shown in Fig. 6(c) and (d), respectively. The former picture presents an early fatigue crack tip. In front of this crack tip, many plastic features in slip bands are concentrated. The succession of local cracks generated in the zones observed in Fig. 6(c) and (d) clearly outlines that the overall damage mechanism is governed by the coalescence of small cracks along these plastic features, in line with the behavior already reported in SLM-processed Ti-6Al-4V alloys [36].

Furthermore, some EBSD images were taken on the lateral surface of a tensile specimen to identify the nature of the plastic features. Therefore, finishing by colloidal

silica suspension was involved in order to prepare a fine lateral surface prior to the tensile testing procedure. To restrict the effect of the final failure on the present observations, this analysis was conducted on regions at least 1 mm away from the specimen fracture surface. The results obtained on two distinct zones are depicted in Fig. 7(a-b) and (c-d), where the first picture shows a SEM micrograph while the second image represents the IPF map superposed with the same SEM micrograph. Per the IPF maps, one can observe intense plastic features along the interface between neighboring elongated primary α-Ti grains. Such plastic features may nucleate a crack in the shear band along the interface between the primary grain and its neighbors, as shown in Fig. 7(c). In addition to the shear bands, the generation of numerous plastic traces spreading over the primary grain thickness can be found in both analyzed regions of Fig. 7. Since these plastic damage traces are limited to the primary α-Ti grains, they are likely related to a crystallographic slip mechanism. The analysis of the angles between seven distinct plastic features (numbered from *b*-① to *b*-⑦) and SEM horizontal axis suggests that the corresponding activate slip systems were exclusively prismatic ones. This trend was confirmed by the analysis of another region in Fig. 7(d). Therefore, there was a high activity of the prismatic slip systems in the primary α-Ti grains during tension of ZTP1 material in horizontal laoding configuration.

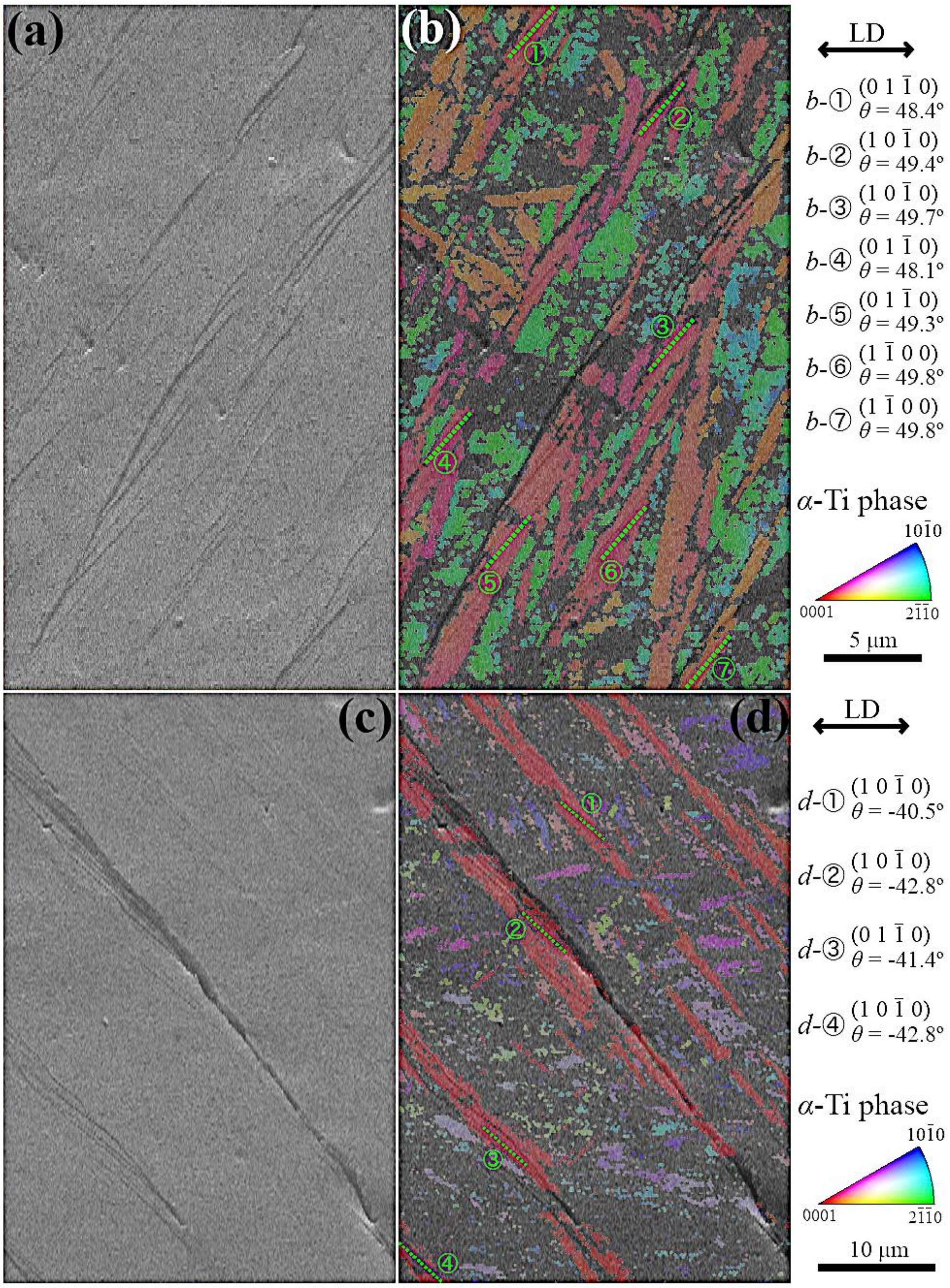

Fig. 7 EBSD analysis of the plastic traces on the lateral surface of ZTP1 specimens subjected to tensile loading. (a) SEM image taken on a specimen strained up to ε = 2.25 %; (b) Superposition of SEM and IPF maps taken on the region shown in (a) (step size: 0.20 μm); (c) SEM image taken on a failed specimen; (d) Superposition of SEM and IPF maps of the zone shown in (c) (step size: 0.25 μm). Only those points are indexed in the IPF image which have a confidence index larger than 0.100.

Table 5. Analysis of the crystallographic orientation of the plastic traces shown in Fig. 7(b) and (d), thereby revealing the type of active slip systems.

Grain	Euler angles (º)			Activated system plane	Schmid factor m	Slip plane trace angle θ (º)	α-grain major axis orientation ζ (º)*
	φ_1	Φ	φ_2				
b-①	271.0	148.2	137.3	$(0\,1\,\bar{1}\,0)$	0.499	48.4	61.8
b-②	268.7	148.2	193.9	$(1\,0\,\bar{1}\,0)$	0.499	49.4	51.8
b-③	268.4	147.9	193.4	$(1\,0\,\bar{1}\,0)$	0.500	49.7	/
b-④	263.0	148.2	309.4	$(0\,1\,\bar{1}\,0)$	0.498	48.1	/
b-⑤	90.6	31.9	224.0	$(0\,1\,\bar{1}\,0)$	0.500	49.3	61.9
b-⑥	86.5	32.0	348.7	$(1\,\bar{1}\,0\,0)$	0.499	49.8	/
b-⑦	98.0	30.4	337.6	$(1\,\bar{1}\,0\,0)$	0.497	49.8	52.1
d-①	295.2	168.6	305.3	$(1\,0\,\bar{1}\,0)$	0.490	-40.5	/
d-②	116.3	13.3	351.4	$(1\,0\,\bar{1}\,0)$	0.494	-42.8	-52.8
d-③	292.3	168.9	63.3	$(0\,1\,\bar{1}\,0)$	0.493	-41.4	-48.3
d-④	112.4	10.6	355.2	$(1\,0\,\bar{1}\,0)$	0.496	-42.8	/

* Calculation withdrawn in the case where the local microstructure is not sufficiently clear.

The characteristics of the activated slip systems identified by EBSD in Fig. 7 are listed in Table 5. Every observed slip activity corresponds to prismatic slip systems with extremely elevated Schmid factor values, $m > 0.490$. Some other works have already outlined the activation of the prismatic slip systems of the primary α-Ti grains in AMed Ti-6Al-4V [45,46]. In addition to the influence of the Schmid factor on slip system activation, Liu et al. [45] have underlined the impact of the spatial orientation of the α-lath traces, where a geometrical orientation generating a high shear stress are likely to activate the plastic slip phenomenon. The spatial orientation was assessed by post-treated EBSD data, providing the angle between the direction of the long axis of α-Ti grain and LD, which will be denoted ζ for the rest of the document. Table 5 shows that primary grains exhibiting severe shear band formation are oriented in directions relatively close to the maximum shear stress orientation $\zeta = 45°$.

3.5. Investigation of the dislocation activity

The investigation of the dislocation activity was carried out by the ECCI method. The related experimental results from two distinct regions are presented in Fig. 8(a) and (c). Each micrograph is completed with the IPF map of the corresponding zones obtained by the EBSD method in Fig. 8(b) and (d). In Fig. 8(a), the GBs of an elongated α-Ti grain are marked by white dotted lines. In this grain, fine white traces parallel to each other are highlighted by solid red lines. From the crystallographic orientation of this grain (see Fig. 8(b)), we calculated the angle between the horizontal direction and the slip trace of the prismatic slip systems with the highest Schmid factor (m = 0.482), and the value of θ = 28.9º was obtained which corresponds to the actual angles observed in the Fig. 8(a). For the sake of exhaustivity, Table S1 of the Supplementary material lists the Schmid factors and plane trace orientations of every considered slip system. In accordance with the orientation of the lines highlighted by ECCI micrographs, the most plausible solution among these 24 systems in each spot is related to the activation of a prismatic slip systems, which are associated to elevated Schmid factors. This agreement suggests that prismatic dislocations glided during tension, resulting in the observed slip traces. Furthermore, several locations highlighted by green ellipses present a clear contrast in Fig. 8(a). Following the ECCI observation principle, this contrast indicates the accumulation of dislocations at these locations. It is interesting to note that the position of the region showing a clear local misorientation, as highlighted by the blue circle in the inset of Fig. 8(b), corresponds well to the high contrast location marked by the blue asterisk in Fig. 8(a). At this position, the accumulation of dislocations is sufficient to generate a local lattice rotation. Furthermore, one can notice that every region showing a contrast is located near a GB.

In the case of a specimen strained in horizontal loading condition (i.e., LD is orthogonal to BD), a model based on the RVE technique by Somlo et al. [47] has predicted that slip localization is found typically in primary α-Ti grains close to the interface with neighboring grains. Moreover, the same model has anticipated the activation of the prismatic slip in the primary α-Ti grains. This result has been confirmed experimentally by transmission electron microscopy (TEM) [45] analysis on an AMed Ti-6Al-4V alloy in which the formation of <*a*>-type dislocations on prismatic slip planes and their accumulation at GBs have been observed.

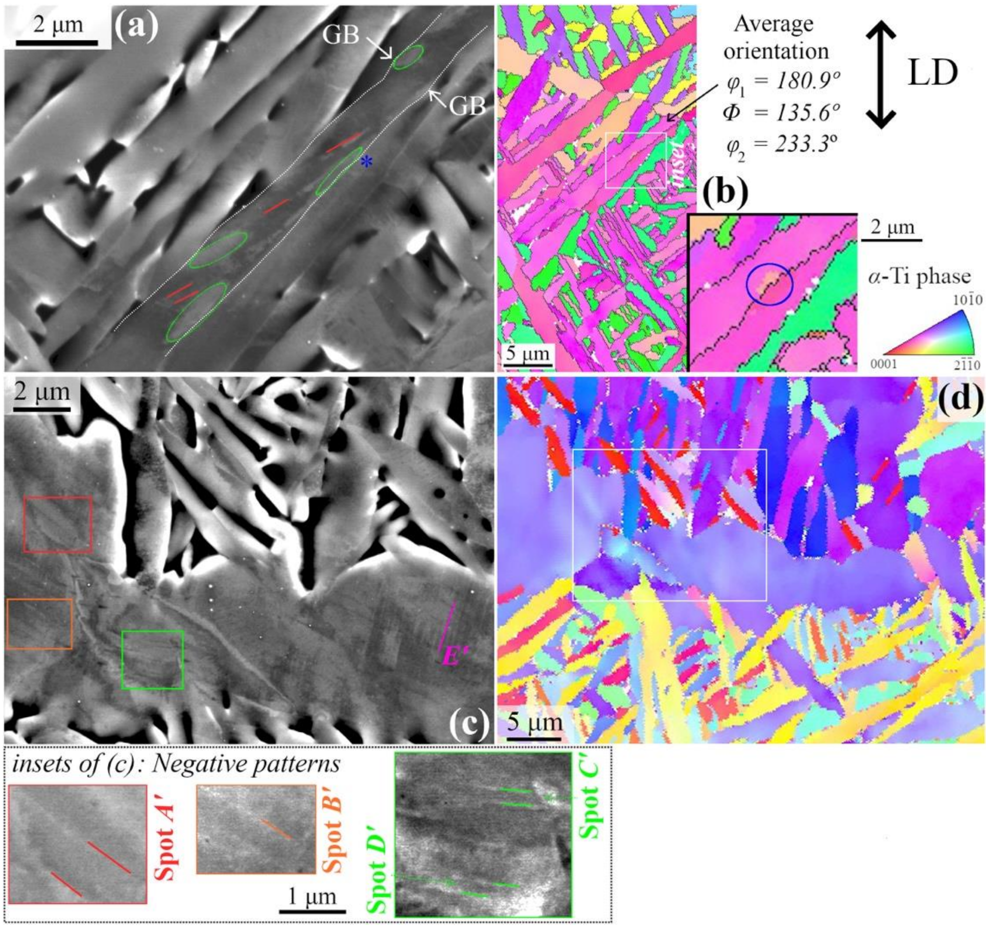

Fig. 8. (a) ECCI micrograph taken on α-Ti grains; (b) IPF map of the region shown in (a) with black lines representing the HAGBs, and the blue circle in the inset indicates the occurrence of a local lattice rotation; (c) ECCI observation on one grain located

between two prior β-grains; and (d) IPF map of the region shown in (c). The insets under (c) show highly magnified micrographs for a better visibility of the locations *A', B', C'* and *D'* analyzed by EBSD.

ECCI study of slip traces was also performed on an equiaxial α-grain formed between two prior β-grains in the region shown in Fig. 8(c). Such equiaxial grains have already been reported for an unreinforced SLMed Ti-6Al-4V alloy [48]. This grain is interesting since the IPF map in Fig. 8(d) reveals some abrupt changes in crystal orientation in its central region, which can induce variation in the orientation of the dislocation traces. Similar to Fig. 8(a), the ECCI micrograph in Fig. 8(c) also presents evident contrast in straight lines. Five different spots (denoted by letters from *A'* to *E'*) were analyzed based on the local crystal orientation obtained by EBSD. The insets under Fig. 8(c) show highly magnified micrographs for a better visibility of the locations *A', B', C'* and *D'* analyzed by EBSD. Based on the ECCI micrographs, the orientations of the traces indicated by solid lines in Fig. 8(c) and its insets are listed in Table 6. The traces observed in the spots from *A'* to *D'* have been analyzed based on the lattice orientation. In accordance with the orientation of the lines highlighted by ECCI micrographs, the most plausible solution among these 24 systems in each spot is related to the activation of a prismatic slip systems associated to relatively elevated Schmid factors (see Table S1 of the Supplementary materials). Furthermore, on the right-hand side of Fig. 8(c), spot *E'* highlights a series of traces related to the activation of basal slip.

Table 6. Analysis of the trace orientations observed in Fig. 8(c).

Region	Euler angles (°)			Detected trace plane	Schmid factor *m*	Plane trace angle θ (°)
	φ_1	Φ	φ_2			
A'	341.1	69.1	169.0	$(1\ \bar{1}\ 0\ 0)$	0.452*	-36.1
B'	340.6	69.0	170.1	$(1\ \bar{1}\ 0\ 0)$	0.451*	-36.1
C'	165.5	104.0	112.5	$(1\ 0\ \bar{1}\ 0)$	0.436*	-4.0
D'	162.3	106.5	356.8	$(1\ \bar{1}\ 0\ 0)$	0.380	-7.2
E'	340.7	68.7	172.3	(0 0 0 1)	0.076~ 0.283	70.7

* largest schmid factor among the prismatic slip systems.

XLPA method was used to study the evolution of the dislocation density during the tensile test of the investigated material. This analysis was carried out on four specimens: (i) initial state before deformation, (ii) tensile tested up to a total strain of 1.00%, (iii) tensile test interrupted at a total strain of 2.25% and (iv) deformed up to failure. The mean crystallite size <*x*>, the average dislocation density ρ and the fraction of β-phase determined for the four samples are listed in Table 7. Due to the detection limit of XLPA caused by the instrumental broadening, the crystallite size values in the initial state and in the samples strained up to 2.25% can only be described as larger than 300 nm. Nevertheless, the failed specimen has a significantly lower crystallite size value than this limit (about 70 nm). It should be noted that the crystallite size determined by XLPA is often smaller than the grain size obtained by microscopic methods (such as EBSD) since the former method is very sensitive to small misorientations. Therefore, in a hierarchical microstructure XLPA gives the subgrain size rather than the grain size [26]. Nevertheless, tensile testing up to the failure resulted in a refinement of the microstructure mainly by subgrain formation.

Tensile straining up to the failure also yielded an order of magnitude increase in the dislocation density. It should be noted that a significant density of dislocations (~0.7 $\times 10^{14}$ m^{-2}) was observed in the AM-processed ZTP1 material even before tensile

testing. These dislocations are grown-in defects that formed during AM processing in order to reduce the mismatch stresses between the misoriented grains nucleated in the laser beam melted layer during building of the ZTP1 samples. In addition, the temperature gradient developed in the specimens due to laser heating during AM processing may cause thermal stresses which can also yield the formation of dislocations.

Table 7. Results of the XLPA method.

Sample	Crystallite size, <x> (nm)	Dislocation density, ρ (10^{14} m^{-2})	Ti β-phase fraction (%)
Initial state	>300	0.7 ± 0.2	4 ± 1
1.00% strained	>300	0.8 ± 0.2	2 ± 1
2.25% strained	>300	2.4 ± 0.4	2 ± 1
Failed	70 ± 9	6 ± 1	5 ± 2

Tension up to the strain of 1 % resulted in only a slight increase in the dislocation density. On the other hand, between 1.00 % and 2.25 % a much larger increase was observed (from ~0.8 × 10^{14} m^{-2} to ~2.4 × 10^{14} m^{-2}). Until the failure (at the strain of about 18 %), this value further increased to ~6 × 10^{14} m^{-2}. In addition to the dislocation density, the XLPA method can also identify the type of dislocations. For every specimen, 80-90 % of dislocations have <*a*>-type Burgers vector. Approximately 60 % of these dislocations glide on a prismatic or pyramidal plane. It should be noted that in XLPA evaluation it is difficult to make a difference between the <*a*>-type dislocations lying on prismatic or pyramidal plane due to their similar effect on X-ray diffraction peak broadening. Thus, the majority of dislocations may be of <*a*>-type prismatic in accordance with the EBSD and ECCI analyses presented in Section 3.4 and above in the present section. XLPA suggested that about 16 % of <*a*>-type dislocations are of basal type in the specimen tested until failure. Therefore, the activity of

dislocations on basal planes observed by the ECCI method is confirmed by XLPA. It is noted that the fraction of <*c*+*a*>-type dislocations increased from 5 to 13 % at the expense of <*a*>-type dislocations with increasing the strain until the failure, suggesting the activation of <*c*+*a*> dislocations in the last stage of the test. XRD analysis also showed that the fraction of β-phase remained practically unchanged during tension, since the scattering of the data in Table 7 can be caused by the slight fluctuation of the phase composition in the specimens cut from different regions of the AM-processed material.

4. Discussion on the hardening mechanism in ZTP1 material

4.1. Increase in the yield stress of Ti-6Al-4V alloy due to nYSZ addition

On the basis of former studies on mechanical properties of MMCs [17,38], the addition of nYSZ to Ti-6Al-4V alloy resulted an increase of the yield stress due to two effects: (i) Orowan hardening owing to the non-coherent interface between the ceramic nano-particles and the metallic matrix and (ii) the addition of zirconia undoubtedly increased the overall oxygen content in the ZTP1 material, leading to solution hardening. Although, the addition of nYSZ was reported to reduce the length of the α-Ti grains [23], the present work suggested no detectable effect of nYSZ on the thickness of the primary grains. Since the Hall-Petch strengthening of materials with lamellar grain morphology is usually computed from the lamella thickness [17,38,49], the addition of 1.0 wt.% nYSZ did not cause a grain refinement sufficient to generate strengthening by Hall-Petch mechanism.

In the case of ZTP1 material, the Orowan strengthening contribution of nYSZ particles to the yield stress, $\Delta\sigma_{or}$, can be obtained through the following equation [50,51]:

$$\Delta\sigma_{or} = \frac{0.4M}{\pi\sqrt{1-\nu}}\frac{Gb}{\lambda}ln\left(\frac{\sqrt{2/3}d}{b}\right), \quad (1)$$

where ν and *G* are the Poisson's ratio and the shear modulus of the matrix (0.310 and 45 GPa [52], correspondingly), *b* is the magnitude of the Burgers vector (0.293 nm, considering that the main slip system is prismatic <*a*>-type), *λ* is the average distance between two adjacent strengthening particles and *d* is the particle diameter. *M* is the Taylor factor, which varies between 2.1 and 4.5 depending on the crystallographic texture and the dominant slip system in the studied HCP material [53]. Thus, in this analysis an average value of 3.3 was selected for the Taylor factor, as a first attempt. Considering the micrograph Fig. 5(b), a first assessment of the average values of *λ* and *d* was carried out, resulting in the numerical values of 270 and 20 nm, respectively. Consequently, the Orowan strengthening should have contributed to the yield stress increase by a value of approximately 99 MPa.

Furthermore, in line with the discussion in Section 3.2, adding nYSZ should have increased the oxygen concentration in the matrix, resulting in a hardening phenomenon through the solid solution effect. A previous work has shown [23], that the same manufacturing process had led to oxygen contents in Ti-6Al-4V and ZTP1 after stress relief heat treatment (HT) of 0.19 and 0.37 wt.%, respectively. Considering that the HIP operated in an inert environment does not influence significantly these concentrations, the yield stress increase, $\Delta\sigma_{SS}$, induced by this solid solution effect can be assessed via the following approach proposed by Choi et al. [17]:

$$\Delta\sigma_{SS} = k_{SS}{C_{\mathrm{O,eq}}}^{n}, \quad (2)$$

where k_{SS} is the strengthening coefficient, $C_{O,eq}$ is the equivalent concentration of soluble O atoms (wt.%), and *n* is the concentration exponent. In accordance with Liu et al. [54], the k_{SS} and *n* have been assessed as 628 MPa/(wt.%)$^{1/2}$ and 0.5, respectively. Furthermore, the equivalent oxygen concentration $C_{O,eq}$, is calculated through oxygen C_O, nitrogen C_N, and carbon C_C concentrations via the following formula: $C_{O,eq} = C_O + 2C_N + 0.75C_C$ [55]. Due to the negligible concentrations of nitrogen (<0.01%) and carbon elements (<0.01%), the equivalent oxygen concentration $C_{O,eq}$ can be taken as the oxygen content solely. Using Eq. (2), the yield stress increase due to the elevated oxygen concentration associated to the addition of 1.0 wt.% nYSZ is evaluated as ~108 MPa.

Moreover, as already mentioned in Section 2.1, the addition of 1.0 % nYSZ has promoted the generation of a strong texture, especially in $(11\bar{2}0)_\alpha$ direction along BD (see Fig. 2(c)). As suggested in our previous work [23], this texture is suspected to influence the tensile properties of the ZTP1 HT+HIP material significantly. Similar to Fang et al. [48], the influence of the texture on the yield stress was studied via a statistical approach based on the grain characteristics of the investigated material. Therefore, the EBSD measurement was performed on a 550 μm × 550 μm area in order to collect data from a representative region of ZTP1 HT+HIP material (the related IPF map is shown in Fig. S2 of the Supplementary material). Based on the definition of the primary and secondary *α*-Ti grains already mentioned in Section 3.1, the orientation of these grains was used to assess the Schmid factor *m* of each slip system considered. According to the experimental results obtained from ECCI and XLPA analyses, most of the dislocation activity should occur on basal and prismatic <*a*> slip systems. Therefore, the present analysis considers exclusively these two system families.

The calculation of the related theoretical axial loading required for activation of each system can be evaluated by $\sigma = \tau_{CRSS} / m$, where τ_{CRSS} is the critical resolved shear stress for the involved slip system family. The present analysis uses the estimation of the critical resolved shear stress τ_{CRSS} reported by Li et al. [56] for Ti-5Al-2.5Sn alloy at room temperature, where the value of the critical resolved shear stresses for the prismatic and basal systems are 0.81 and 1.00, respectively, since the critical resolved shear stress for basal systems is considered as the reference. Therefore, for each examined grain the uniaxial stress necessary for the activation of basal and prismatic slip systems (referred to as "*YS.B*" and "*YS.P*", respectively) were assessed as the minimum stress over the three distinct systems of each family. Then, the overall average strength values over the whole material (denoted as $YS.B_{avg}$ and $YS.P_{avg}$) were determined by considering the grain area as the weighting factor. Furthermore, the assessment of the area where such estimated uniaxial stress (*YS*) is minimal for a basal or a prismatic slip system are denoted as "*Area.B*" and "*Area.P*", respectively. The results of this analysis are shown in Table 8, where two cases were taken into consideration: (i) horizontal and (ii) vertical loading configurations (i.e., LD ⊥ BD and LD // BD, respectively). This analysis was carried out on primary grains exclusively and on both primary and secondary grains.

Table 8. Analysis of basal (B) and prismatic (P) slip system activity via EBSD data.

Loading	Horizontal		Vertical	
Grain considered	Exclusively Primary	Primary & Secondary	Exclusively Primary	Primary & Secondary
$YS.B_{avg}$	5.65	5.85	17.9	17.3
$YS.P_{avg}$	4.63	4.35	3.26	3.77
AreaB (%)	43.8	43.8	39.3	46.4
AreaP (%)	56.2	56.2	60.7	53.6

In the vertical loading configuration, the average strength values for basal slip systems ($YS.B_{avg}$) are approximately five times larger than the corresponding ones for the prismatic systems (i.e., $YS.P_{avg}$). The high value of $YS.B_{avg}$ is explained by the notably high magnitude at the extremity of the $(0002)_{\alpha}$ pole figure (see Fig. 2(c)). This property is an outcome of the strong $(11\bar{2}0)_{\alpha}$ fiber, as represented in Fig. 9(a) and (b). Therefore, as underlined by Fig. 9(a), whatever the orientation of the lattice obeying the this $(11\bar{2}0)_{\alpha}$ fiber, the basal plane normal $\overrightarrow{n_B}$ is orthogonal to A3. Therefore, in the case of the vertical loading (i.e, LD is identical to A3), any of the grain presenting such an orientation results in an unfavorable activation of any basal slip system. On the other hand, under horizontal loading, Schmid factor of such grains can reach various values, and possibly large ones if the angle between the basal plane normal and the LD is close to 45º. As a result, the large amount of unfavorably oriented grains for basal slip in vertical loading impacts significantly the average value $YS.B_{avg}$. Nevertheless, the fraction of area where axial stress is minimal for basal slip systems *Area.B* is 46.4 %.

In addition to the plummet of the strength related to the basal slip systems *YS.B*, it should be noted that the *YS.P* also tends to increase in horizontal loading condition by 30% with respect to the corresponding value in vertical loading configuration. As represented in Fig. 9(a), considering the case where the $(11\bar{2}0)_{\alpha}$ pole is exactly at the figure center, irrespective to the position of A1 and A2 axes, two prismatic slip systems presents large Schmid factor values. Indeed, the angle between the prismatic slip plane normal and the axis A3 is 30º, and the angle between the plane slip direction and A3 is 60º. Therefore, considering vertical loading configuration, any of the grain constitutive to the $(11\bar{2}0)_{\alpha}$ fiber presents a maximal Schmid factor close to 0.433 (= cos(π/6)cos(π/3)) among prismatic systems. Conversely, the case of the horizontal loading will results in a large range of Schmid factor value from zero (LD orthogonal to

prismatic slip plane normal $\overrightarrow{n_{PR}}$) to a maximual one of 0.433 ($\overrightarrow{n_{PR}}$ included into LD-A3 plane). For this reason, the average strength governed by the prismatic slip *YS.P* underlines larger values in the horizontal loading configuration than in the vertical one.

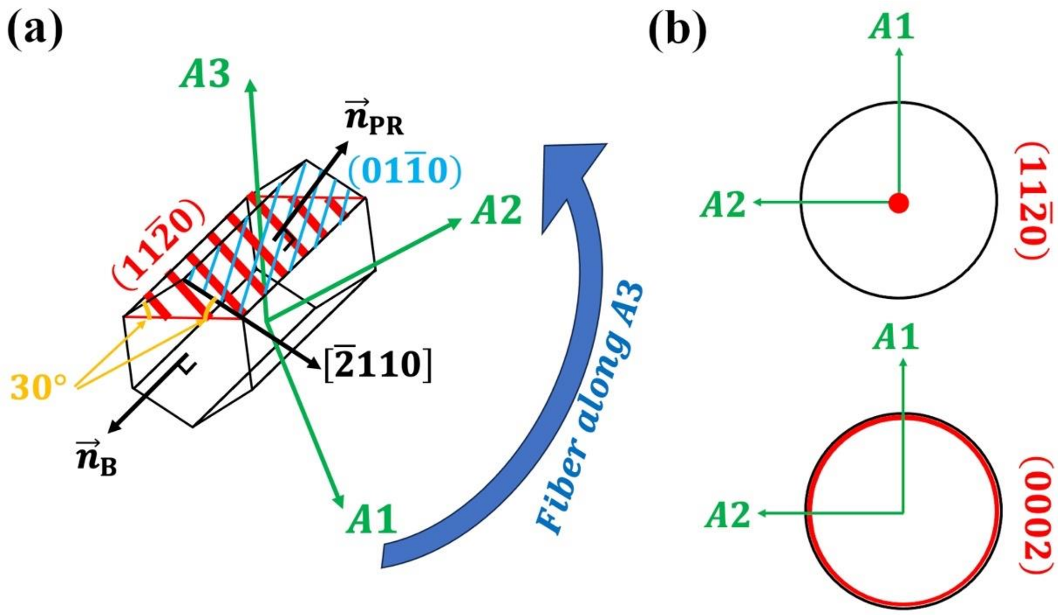

Fig. 9 (a) Representation of the $(11\bar{2}0)_\alpha$ fiber along the A3 direction; (b) theoretical pole figure generated by this fiber for $(11\bar{2}0)_\alpha$ and $(0002)_\alpha$ planes.

The outcomes from this procedure underlines the importance of the basal slip in the tensile behavior of the investigated material. Despite the nucleation of plastic features along the prismatic slip systems of the α-Ti primary grains in horizontal loading configuration, this result suggests that the large compressive strength of ZTP1 HT+HIP material found under vertical loading in [23] is related to the large stress required for basal slip activation. This is in line with the discussions undertaken by Somlo et al. [47], where an RTE model has pointed out that the activation of basal slip mainly governs the plastic features of AMed Ti-6Al-4V for a vertical loading

configuration. Accordingly, the investigated ZTP1 HT+HIP material should present a strong strength anisotropy.

4.2. Strain hardening behavior

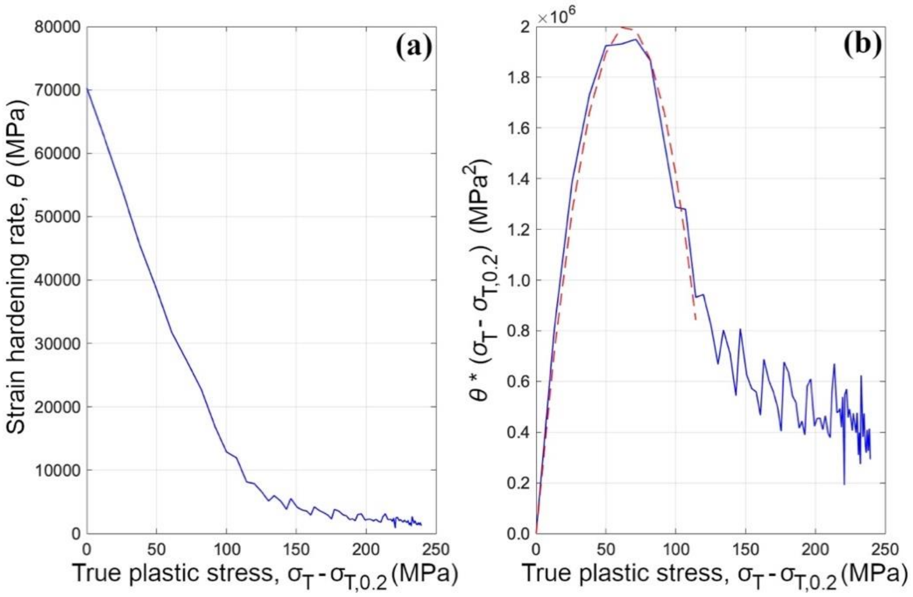

Fig. 10. (a) Strain hardening rate versus the true plastic stress for ZTP1 HT+HIP material; (b) Fluctuation of the product of strain hardening rate and the true plastic stress as a function of the true plastic stress. In (b), the dashed curve represents a fitting on the experimental data using Eq (4).

In addition to the increase of the yield stress due to the addition of nYSZ, Section 3.2 has underlined a strong strain hardening capacity of ZTP1 HT+HIP material. In line with former studies [48,57–59], the strain hardening rate $\theta = d\sigma/d\varepsilon$ versus the true plastic strain σ_T-$\sigma_{T,0.2}$ is plotted in Fig. 10(a). A steady decreasing hardening rate, extended up to the true plastic stress of approximately 120 MPa, corresponds to the plastic deformation stage II. Then, stage III occurs in a relatively extended true plastic strain range between 120 and 240 MPa; the latter stress is related

to the onset of necking. Since the strain hardening phenomenon is intrinsically related to the dislocation behavior, Lukac and Balik [60] have expressed the variation of the strain hardening rate by (i) hardening phenomena caused by the multiplication of dislocations at impenetrable obstacles and forest dislocations and (ii) softening phenomena imposed by dislocation annihilation by cross-slip and dislocation climb. This approach leads to the following expression of the strain hardening rate θ for polycrystal materials [60]:

$$\theta = \frac{A_H}{\sigma_{\mathrm{T}}-\sigma_{\mathrm{T,0.2}}} + B_H - C_H(\sigma_{\mathrm{T}} - \sigma_{\mathrm{T,0.2}}) - D_H(\sigma_{\mathrm{T}} - \sigma_{\mathrm{T,0.2}})^3, \quad (3)$$

where the parameter A_{H} represents the interaction of dislocations with non-dislocation obstacles (such as precipitates), the parameter B_{H} is connected with strain hardening induced by dislocation forests, the parameter C_{H} is related to strain softening due to cross-slip of screw dislocations, and the parameter D_{H} highlights the effect of dislocation climb. In a way similar to the analysis of the strain-hardening phenomenon in HCP Mg-alloys operated by Qiao et al. [61], the straightforward relation between $\theta\times(\sigma_{\mathrm{T}}-\sigma_{\mathrm{T,0.2}})$ and $\sigma_{\mathrm{T}}-\sigma_{\mathrm{T,0.2}}$ expressed in Eq. (4) is considered.

$$\theta(\sigma_{\mathrm{T}} - \sigma_{\mathrm{T,0.2}}) = A_H + B_H(\sigma_{\mathrm{T}} - \sigma_{\mathrm{T,0.2}}) - C_H(\sigma_{\mathrm{T}} - \sigma_{\mathrm{T,0.2}})^2 - D_H(\sigma_{\mathrm{T}} - \sigma_{\mathrm{T,0.2}})^4 \quad (4)$$

The parameters of this equation can be estimated via the analysis of the product $\theta\times(\sigma_{\mathrm{T}}-\sigma_{\mathrm{T,0.2}})$ versus the true plastic stress $\sigma_{\mathrm{T}}-\sigma_{\mathrm{T,0.2}}$, as shown in Fig. 10(b). The smooth evolution of this curve close to null stress values suggests that the influence of the parameter A_{H} can be neglected as a first approximation. The limited influence of this parameter on the overall strain hardening suggests the low strain hardening effect of the nYSZ particles during tension, although they have a non-negligeable influence on the

yield stress. Furthermore, it can be assumed that the strain hardening at room temperature of ZTP1 material is not affected significantly by the climb of dislocations; therefore, $D_H = 0$ was selected. In accordance with these assumptions, Eq. (4) is reduced to a parabolic function determined by B_H and C_H parameters, which reflects dislocation multiplication and annihilation, respectively. A fitting on the experimental curve in the strain hardening stage II was performed, which is shown by a dashed red line in Fig. 10(b). The apparent good agreement between the experimental data and the fitting curve confirms that the hardening rate is influenced essentially by dislocation forest generation and cross-slip mechanisms. Experimental values of parameters B_H and C_H are 6.17×10^4 MPa and 475, respectively. The soluble oxygen element into the α-Ti matrix should contribute significantly in the strong hardening capacity of the investigated material in this stage II.

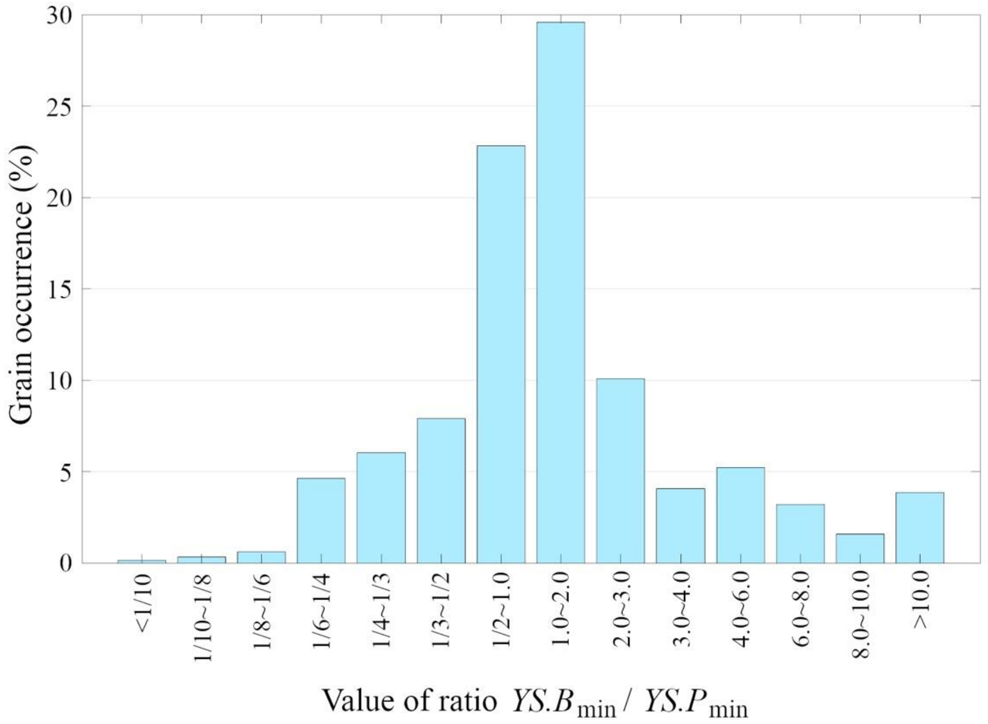

Fig. 11. Distribution of the ratio of the theoretical minimal critical loading of basal system over the prismatic ones for primary and secondary α-Ti grains.

In a former paper, Del Valle et al. [62] have outlined that the extension of the hardening stage III is caused by an intense cross-slip activity in magnesium-based HCP alloys. Such cross-slip activities are promoted by the increasing resolved shear stress of the activated slip systems in the strain-hardening stage II. In line with the experimental results obtained from ECCI and XLPA techniques (see Section 3.5), the majority of dislocations should be of basal and pyramidal <*a*>-types. To assess the feasibility of the cross-slip activity between these slip systems in the investigated material, a statistical approach based on the same EBSD data analyzed in Section 4.1 was carried out as follows: considering the actual LD orientation (i.e., horizontal direction) for each primary or secondary α-Ti grain, the ratio of the minimum stress required for plastic slip in basal system $YS.B_{\mathrm{min}}$ over the one for prismatic system $YS.P_{\mathrm{min}}$ was computed. The distribution of the obtained ratio $YS.B_{\mathrm{min}}$ / $YS.P_{\mathrm{min}}$ is represented via the bar diagram in Fig. 11. This distribution underlines a fairly centered characteristic with a mean value close to 1.0. This value corresponds to the theoretical configuration where the occurrence of the plastic slip in basal and prismatic systems have equal feasability. In other words, this configuration favors cross-slip mechanism between basal and prismatic systems. A more refined analysis of the distribution reveals that 52.4% of the considered grains present a ratio between 0.5 and 2.0. This fraction reaches 85.3% if a ratio range between 0.33 and 3.0 is considered. Therefore, the texture of the presently investigated material is likely to promote cross-slip dislocation activity in a large number of primary and secondary α-Ti grains, especially when the activation of “easy” slip systems in the early stage II tends to increase their own resolved shear stresses. This configuration results in two main consequences: (i) a quick decrease of the strain hardening rate in late stage II by dislocation annihilation leading to an early transition to

stage III (see Eq. (4)); and (ii) the extension of the strain hardening stage III by dynamic recovery [62]. Finally, the increasing activity of the pyramidal <*a*+*c*> slip system in the last stage of hardening detected by XLPA (see Section 3.5) is also certainly related to a cross-slip mechanism. Indeed, despite their elevated critical resolved shear stress (i.e., 35 times larger than for the basal system [56]), which strongly limit their activation in the strain hardening stage II, activation of favorably oriented pyramidal <*a*+*c*> systems by cross-slip is reasonable in stage III.

5. Conclusions

In the present work, the tensile behavior of SLM-processed Ti-6Al-4V alloy reinforced with 1 wt.% nYSZ was investigated. Various aspects related to the tensile straining were analyzed, such as the cracking behavior, quantification of the yield stress increase, characterization of the dislocation activity and interpretation of the strain-hardening behavior. The most important results can be summarized as follows:

1. Adding 1 wt.% nYSZ resulted in the formation of nano-sized particles containing yttrium element. Even though this addition reduced the length of α-Ti laths, the primary α-Ti lath thickness remained unchanged, resulting in no strengthening by Hall-Petch mechanism.

2. Under horizontal loading direction, ZTP1 HT+HIP material possesses a yield stress and tensile strength values of 999 and 1195 MPa, respectively. Such values are notably higher than its unreinforced counterparts after HIP post-processing. Furthermore, the investigated material presents a strong ability for strain hardening.

3. Early cracking occurs along primary α-Ti GBs which was accompanied by the activation of prismatic slip systems. This result is similar to its AMed unreinforced Ti-6Al-4V counterparts.

4. The effects of dispersion hardening and solute oxygen strengthening caused by the addition of nYSZ particles were similar (i.e., about 100 MPa for each). Furthermore, a statistical approach was applied to determine the effect of the texture, which forecasts a strong yield stress anisotropy. Therefore, even more elevated tensile properties are expected for ZTP1 HT+HIP strained in vertical loading configuration (i.e., along the building direction).

5. The addition of soluble oxygen in the matrix should promote the dislocation accumulation and annihilation in strain hardening stage II. Finally, the elongated stage III is induced by the texture of the investigated material, which favors the cross-slip occurring mainly between basal and prismatic slip systems.

Acknowledgments

The support by the Université Sorbonne Paris Nord (USPN) “CARAMEL” platform facility funded by Région Île-de-France and the CNRS for the HIP post-treatments is very welcomed.

Funding

This research did not receive any specific grant from funding agencies in the public, commercial, or not-for-profit sectors.

Data availability

The raw/processed data required to reproduce these findings cannot be shared at this time as the data also forms part of an ongoing research project.

Authors contributions

Benjamin Guennec: Methodology, Formal analysis, Software, Data Curation, Investigation, Visualization; Writing – Original Draft; **Amine Hattal**: Investigation; **Kentaro Nagano:** Investigation. **Azziz Hocini:** Validation. **Kamilla Mukhtarova:** Investigation; **Takahiro Kinoshita:** Supervision. **Noriyo Horikawa**: Supervision, Ressources. **Hiroshi Fujiwara:** Supervision, Ressources; **Jenő Gubicza:** Validation, Writing – Review & Editing; **Madjid Djemaï:** Ressources; **Guy Dirras:** Conceptualization, Project administration; Writing – Review & Editing.

REFERENCES:

[1] C. Emmelmann, P. Sander, J. Kranz, E. Wycisk, Laser Additive Manufacturing and Bionics: Redefining Lightweight Design, Phys. Proc. 12 (2011) 364–368. https://doi.org/10.1016/j.phpro.2011.03.046

[2] D. Herzog, V. Seyda, E. Wycisk, C. Emmelmann, Additive manufacturing of metals, Acta Mater. 117 (2016) 371–392. https://doi.org/10.1016/j.actamat.2016.07.019

[3] N. Khanna, K. Zadafiya, T. Patel, Y. Kaynak, R.A.R. Rashid, A. Vafadar, Review on machining of additively manufactured nickel and titanium alloys, J. Mater. Res. Technol. 15 (2021) 3192–3221. https://doi.org/10.1016/j.jmrt.2021.09.088

[4] T.S. Tshephe, S.O. Akinwamide, E. Olevsky, P.A. Olubambi, Additive manufacturing of titanium-based alloys- A review of methods, properties, challenges, and prospects, Heliyon 8 (2022) e09041. https://doi.org/10.1016/j.heliyon.2022.e09041

[5] H. Monteiro, G. Carmona-Aparicio, I. Lei, M. Despeisse, Energy and material efficiency strategies enabled by metal additive manufacturing – A review for the aeronautics and aerospace sectors, Energy Reports 8 (2022) 298–305. https://doi.org/10.1016/j.egyr.2022.01.035

[6] L. Xiao, W.J. Lu, J.N. Qin, Y.F. Chen, D. Zhang, M.M. Wang, F. Zhu, B. Ji, Steady-state creep of in situ TiB plus La_2O_3 reinforced high temperature titanium matrix composite, Mater. Sci. Eng. A 499 (2009) 500–506. https://doi.org/10.1016/j.msea.2008.09.002

[7] M.Y. Koo, J.S. Park, M.K. Park, K.T. Kim, S.H. Hong, Effect of aspect ratios of in situ formed TiB whiskers on the mechanical properties of TiBw/Ti–6Al–4V

composites, Scr. Mater. 66 (2012) 487–490. https://doi.org/10.1016/j.scriptamat.2011.12.024

[8] S. Pouzet, P. Peyre, C. Gorny, O. Castelnau, T. Baudin, F. Brisset, C. Colin, P. Gadaud, Additive layer manufacturing of titanium matrix composites using the direct metal deposition laser process, Mater. Sci. Eng. A 677 (2016) 171-181. https://doi.org/10.1016/j.msea.2016.09.002

[9] H. Attar, M. Bonisch, M. Calin, L.C. Zhang, K. Zhuravleva, A. Funk, S. Scudino, C. Yang, J. Eckert, Comparative study of microstructures and mechanical properties of in-situ Ti-TiB composites produced by selective laser melting, powder metallurgy, and casting technologies, J. Mater. Res. 29 (2014) 1941-1950. http://doi.org/10.1557/jmr.2014.122

[10] Z.W. Yang, L.Q. Fu, S.L. Wang, M. Zhang, Y. Wang, Z.Q. Ma, D.P. Wang, Balance of strength and plasticity of additive manufactured Ti-6Al-4V alloy by forming TiB whiskers with gradient distribution, Addit. Manuf. 39 (2021) 101883. https://doi.org/10.1016/j.addma.2021.101883

[11] H. Chen, K. Kosiba, T. Lu, N. Yao, Y. Liu, Y. Wang, K.G. Prashanth, C. Suryanarayama, Hierarchical microstructures strengthening mechanisms of nano-TiC reinforced CoCrFeMnNi high-entropy alloy composites prepared by laser powder bed fusion, J. Mater. Sci. Technol. 136 (2023) 245-259. https://doi.org/10.1016/j.jmst.2022.06.053

[12] G. Sivakumar, V. Ananthi, S. Ramanathan, Production and mechanical properties of nano SiC particle reinforced Ti–6Al–4V matrix composite, T. Nonferr. Metal. Soc. 27 (2017) 82–90. https://doi.org/10.1016/S1003-6326(17)60009-8

[13] S. Wen, K. Chen, W. li, Y. Zhou, Q. Wei, Y. Shi, Selective laser melting of reduced graphene oxide/S136 metal matrix composites with tailored microstructures and mechanical properties, Mat. Des. 175 (2019) 107811. https://doi.org/10.1016/j.matdes.2019.107811

[14] B. Song, Z. Wang, Q. Yan, Y. Zhang, J. Zhang, C. Cai, Q. Wei, Y. Shi, Integral method of preparation and fabrication of metal matrix composite: selective laser melting of in-situ nano/submicro-sized carbides reinforced iron matrix composites, Mat. Sci. Eng. A 707 (2017) 478-487. https://doi.org/10.1016/j.msea.2017.09.092

[15] X.B. Zhou, J.T.M. De Hosson, Reactive wetting of liquid metals on ceramic substrates, Acta Mater. 44 (1996) 421-426. https://doi.org/10.1016/1359-6454(95)00235-9

[16] S. Jayalakshmi, M. Gupta, Light metal matrix composites, in: Metallic amourphous alloy reinforcements in light metal matrices, Springer International publishing, Cham, 2015 7-58. https://doi.org/10.1007/978-3-319-15016-1

[17] G. Choi, W.S. Choi, Y.S. Lee, D. Kim et al., Decomposition behavior of yttria-stabilized zirconia and its effect on directed energy deposited Ti-based composite material, J. Mat. Sci. Technol. 112 (2022) 138-150. https://doi.org/10.1016/j.jmst.2021.09.052

[18] H. Conrad, Effect of interstitial solutes on the strength and ductility of titanium, Prog. Mater. Sci. 26 (1981) 123-403. https://doi.org/10.1016/0079-6425(81)90001-3

[19] Z.Z. Fang, J.D. Paramore, P. Sun, K.S.R. Chandran, Y. Zhang, Y. Xia, F. Cao, M. Koopman, Powder metallurgy of titanium – past, present, and future, M. Free, Int. Mater. Rev. 63 (2018) 407-459. https://doi.org/10.1080/09506608.2017.1366003

[20] L.B.O. Madelung (Ed.), Ti-Y (Titanium-Yttrium) Phase Diagram, 1990.

[21] A. Hattal, T. Chauveau, M. Djemai, J.J. Fouchet, B. Bacroix, G. Dirras, Effect of nano-yttria stabilized zirconia addition on the microstructure and mechanical properties of Ti6Al4V parts manufactured by selective laser melting, Mater. Des. 180 (2019) 107909. https://doi.org/10.1016/j.matdes.2019.107909

[22] A. Hattal, M. Djemai, J.J. Fouchet, T. Chauveau, B. Bacroix, A. Hocini, G. Dirras, Titanium Ti6Al4V alloy reinforced by the addition of nano yttria stabilized zirconia fabricated by selective additive manufacturing: microstructure and mechanical investigations, MATEC Web Conf. 321 (2020) 03018. https://doi.org/10.1051/matecconf/202032103018

[23] A. Hattal, K. Mukhtarova, M. Djemai, T. Chauveau, A. Hocini, J.J. Fouchet, B. Bacroix, J. Gubicza, G. Dirras, Effect of hot isostatic pressing on microstructure and mechanical properties of Ti6Al4V-zirconia nanocomposites processed by laser-powder bed fusion, Mater. Des. 214 (2022) 110392. https://doi.org/10.1016/j.matdes.2022.110392

[24] B. Guennec, A. Hattal, A. Hocini, K. Mukhtarova, T. Kinoshita, N. Horikawa, J. Gubicza, M. Djemaï, G. Dirras, Fatigue performance of zirconia-reinforced Ti-6Al-4V nanocomposite by laser powder bed fusion: An improvement by hot isostatic pressing, Int. J. Fatigue, 164 (2022) 107129. https://doi.org/10.1016/j.ijfatigue.2022.107129

[25] K. Mergia, K.L. Stefanopoulos, N. Ordás, C. García-Rosales, A comparative study of the porosity of doped graphites by small angle neutron scattering, nitrogen adsorption

and helium pycnometry, Microporous and Mesoporous Materials. 134 (2010) 141–149. https://doi.org/10.1016/j.micromeso.2010.05.019

[26] J. Gubicza, X-ray line profile analysis in Materials Science, IGI-Global, Hershey, PA, USA.

[27] G. Ribarik, J. Gubicza, T. Ungar, Correlation between strength and microstructure of ball-milled Al-Mg alloys determined by X-ray diffraction, Mat. Sci. Eng. A 387–389 (2004) 343–347. https://doi.org/10.1016/j.msea.2004.01.089

[28] B. Vrancken, L. Thijs, J.-P. Kruth, J. Van Humbeeck, Heat treatment of Ti6Al4V produced by Selective Laser Melting: Microstructure and mechanical properties, Journal of Alloys and Compounds. 541 (2012) 177–185. https://doi.org/10.1016/j.jallcom.2012.07.022

[29] D. Greitmeier, F. Palm, F. Syassen, T. Melz, Fatigue performance of additive manufactured TiAl64V using electron and laser beam melting, Int. J. Fatigue 94 (2017) 211-217. https://doi.org/10.1016/j.ijfatigue.2016.05.001

[30] E. Wycisk, S. Siddique, D. Herzog, F. Walther, C. Emmelmann, Fatigue performance of laser additive manufactured Ti-6Al-4V in very high cycles fatigue regime up to 10^9 cycles, Front. Mater. 2 (2015) 72. https://doi.org/10.3389/fmats.2015.00072

[31] K. Chang, X. Wang, E. Liang, R. Zhang, On the texture and mechanical property anisotropy of Ti6Al4V alloy fabricated by powder-bed based laser additive manufacturing, Vacuum 181 (2020) 109732. https://doi.org/10.1016/j.vacuum.2020.109732

[32] M. Fang, F. Hu, Y. Han, J. Le, J. Xi, J. Song, L. Ke, M. Xiao, W. Lu, Controllable mechanical anisotropy of selective laser melted Ti6Al4V: A new perspective into the effect of grain orientations and primary grain structure, Mat. Sci. Eng. A 827 (2021) 142031. https://doi.org/10.1016/j.msea.2021.142031

[33] S. Lu, Z.J. Zhang, R. Liu, X.H. Zhou, X.G. Wang, B.N. Zhang, X.M. Zhao, J. Eckert, Z.F. Zhang, Optimal tensile properties of laser powder bed fusion hereditary basket-weave microstructure in additive manufactured Ti6Al4V, Additive manufacturing 59 (2022) 103092. https://doi.org/10.1016/j.addma.2022.103092

[34] S. Leuders, M. Thone, A. Riemer, T. Niendorf, T. Troster, H.A. Richard, H.J. Maier, On the mechanical behaviour of titanium alloy TiAl64V manufactured by selective laser melting: Fatigue resistance and crack growth performance, Int. J. Fatigue 48 (2013) 300-307. https://doi.org/10.1016/j.ijfatigue.2012.11.011

[35] G. Kasperovich, J. Hausmann, Improvement of fatigue resistance and ductility of TiAl6V4 processed by selective laser melting, J. Mat. Proc. Tec. 220 (2015) 202-214. https://doi.org/10.1016/j.jmatprotec.2015.01.025

[36] A. Moridi, A.G. Demir, L. Caprio, A.J. Hart, B. Previtali, B.M. Colosimo, Deformation and failure mechanism of Ti-6Al-4V as built by selective laser melting, Mat. Sci. Eng. A 768 (2019) 138456. https://doi.org/10.1016/j.msea.2019.138456

[37] X.P. Ren, H.Q. Li, H. Guo, F.L. Shen, C.X. Qin, E.T. Zhao, X.Y. Fang, A comparative study on mechanical properties of Ti-6Al-4V alloy processed by additive manufacturing vs. traditional processing, Mat. Sci. Eng. A 817 (2021) 141384. https://doi.org/10.1016/j.msea.2021.141384

[38] X. Wang, L.-J. Zhang, J. Ning, S. Li, L.-L. Zhang, J. Long, Hierarchical grain refinement during laser additive manufacturing of Ti-6Al-4V alloys by the addition of micro-sized refractory particles, Additive Manufacturing 45 (2021) 102045. https://doi.org/10.1016/j.addma.2021.102045

[39] Failure mechanisms data analysis during tension of additively manufactured Ti-6Al-4V alloy reinforced with nano-zirconia particles: Investigations of the crack path, Data in Brief, under review.

[40] Z.W. Yang, L.Q. Fu, M. Zhang, Y. Wang, Z.Q. Ma, D.P. Wang, Balance of strength and plasticity of additive manufactured Ti-6Al-4V alloy by forming TiB whiskers with cyclic gradient distribution, Additive Manufacturing 39 (2021) 101883. https://doi.org/10.1016/j.addma.2021.101883

[41] H. Li, Z. Yang, D. Cai, D. Jia, Y. Zhou, Microstructure evolution and mechanical properties of selective laser melted bulk-form titanium matrix nanocomposites with minor B_4C additions, Mat. Des. 185 (2020) 108245. https://doi.org/10.1016/j.matdes.2019.108245

[42] C.W. Park, J.M. Byun, W.J. Choi, S.Y. Lee, Y.D. Kim, Improvement of high temperature mechanical properties of Ni-based oxide dispersion strengthened alloys by preferential formation of Y-Ti-O complex oxide, Mat. Sci. Eng. A 740-741 (2019) 363-367. https://doi.org/10.1016/j.msea.2018.10.004

[43] T. Mukherjee, W. Zhang, T. DebRoy, An improved prediction of residual stresses and distortion in additive manufacturing, Comput. Mater. Sci. 126 (2017) 360-372. https://doi.org/10.1016/j.commatsci.2016.10.003

[44] Q. Guo, C. Zhao, M. Qu, L. Xiong, S.M.H. Hojjatzadeh, L.I. Escano, N.D. Parab, K. Fezzaa, T. Sun, L. Chen, In-situ full field mapping of melt flow dynamics in laser

metal additive manufacturing, Additive Manufacturing 31 (2020) 100939. https://doi.org/10.1016/j.addma.2019.100939

[45] Z. Liu, Z. Zhao, J. Liu, Q. Wang, Z. Guo, Y. Zeng, G. Yang, S. Gong, Effects of the crystallographic and spatial orientation of α lamellae on the anisotropic in-situ tensile behaviors of additive manufactured Ti-6Al-4V, J. All. Comp. 850 (2021) 156886. https://doi.org/10.1016/j.jallcom.2020.156886

[46] J. Liu, K. Zhang, X. Gao, H. Wang, S. Wu, Y. Yang, Y. Zhu, A. Huang, Effects of the morphology of grain boundary α-phase on the anisotropic deformation behaviors of additive manufactured Ti-6Al-4V, Mat. Des. 223 (2022) 111150. https://doi.org/10.1016/j.matdes.2022.111150

[47] K. Somlo, K. Poulios, C.V. Funch, C.F. Niordson, Anisotropic tensile behaviour of additively manufactured Ti-6Al-4V simulated with crystal plasticity, Mech. Mater. 162 (2021) 104034. https://doi.org/10.1016/j.mechmat.2021.104034

[48] M. Fang, F. Hu, Y. Han, J. Le, J. Xi, J. Song, L. Ke, M. Xiao, W. Lu, Controllable mechanical anisotropy of selective laser melted Ti6Al4V: A new perspective into the effect of grain orientations and primary grain structure, Mat. Sci. Eng. A 827 (2021) 142031. https://doi.org/10.1016/j.msea.2021.142031

[49] W. Xu, M. Brandt, S. Sun, J. Elambasseril, Q. Liu, K. Latham, K. Xia, M. Qian, Additive manufacturing of strong and ductile Ti-6Al-4V by selective laser melting via in situ martensite decomposition, Acta Mater. 85 (2015) 74-84. https://doi.org/10.1016/j.actamat.2014.11.028

[50] L.M. Brown, R.K. Ham, Strengthening methods in crystals, A. Kelly and R.B. Nicholson, eds., Elsevier, Amsterdam, 1971, pp.9-135.

[51] H.-S. Kim, S.-H. Park, Dissolution-induced oxide synthesis using SiO2 microparticles for oxide dispersion strengthening of Fe-based alloys, J. Mater. Res. Technol. 27 (2023) 449-461. https://doi.org/10.1016/j.jmrt.2023.09.233

[52] M.V. Pantawane, T. Yang, Y. Jin, S.S. Joshi, S. Dasari, A. Sharma, A. Krokhin, S.G. Srinivasan, R. Banerjee, A. Neogi, N.B. Dahotre, Crystallographic texture dependent bulk anisotropic elastic response of additively manufactured Ti6Al4V, Sci. Rep. 11 (2021) 633. https://doi.org/10.1038/s41598-020-80710-6

[53] C.H. Caceres, P. Lukac, Strain hardening behaviour and the Taylor factor of pure magnesium, Philosophical Magazine 88 (2008) 977–989. https://doi.org/10.1080/14786430801968611

[54] Z. Liu, G. Welsch, Effects of the oxygen and heat treatment on the mechanical properties of alpha and beta titanium alloys, Metall. Trans. A 19 (1988) 527-542. https://doi.org/10.1007/BF02649267

[55] H. Conrad, Effect of interstitial solutes on the strength and ductility of titanium, Prog. Mater. Sci. 26 (1981) 123-403. https://doi.org/10.1016/0079-6425(81)90001-3

[56] H. Li, D.E. Mason, T.R. Bieler, C.J. Boehlert, M.A. Crimp, Methodology for estimating the critical resolved shear stress ratios of α-phase Ti using EBSD-based trace analysis, Acta Mater. 61 (2013) 7555-7567. https://doi.org/10.1016/j.actamat.2013.08.042

[57] L. Guo, Z. Chen, L. Gao, Effects of grain size, texture and twinning on mechanical properties and work-hardening behavior of AZ31 magnesium alloys, Mat. Sci. Eng. A 578 (2011) 8537-8545. https://doi.org/10.1016/j.msea.2011.07.076

[58] Y. Qiao, X. Wang, Z. Liu, E. Wang, Effects of grain size, texture and twinning on mechanical properties and work-hardening behaviors of pure Mg, Mat. Sci. Eng. A 578 (2013) 240-246. https://doi.org/10.1016/j.msea.2013.04.094

[58] K. Chang, X. Wang, E. Liang, R. Zhang, On the texture and mechanical property anisotropy of Ti6Al4V alloy fabricated by powder-bed based laser additive manufacturing, Vacuum 181 (2020) 109732. https://doi.org/10.1016/j.vacuum.2020.109732

[60] P. Lukac, J. Balik, Kinetics of plastic deformation, Key Eng. Mater. 97/98 (1994) 307-321. https://doi.org/10.4028/www.scientific.net/KEM.97-98.307

[61] Y. Qiao, X. Wang, Z. Liu, E. Wang, Effect of grain size, texture and twinning on mechanical properties and work-hardening behaviors of pure Mg, Mat. Sci. Eng. A 578 (2013) 240-246.

[62] J.A. Del Valle, F. Carreno, O.A. Ruano, Influence of texture and grain size on work hardening and ductility in magnesium-based processed by ECAP and rolling, Acta Mater. 54 (2006) 4247-4259. https://doi.org/10.1016/j.actamat.2006.05.018

Declaration of interests

☒The authors declare that they have no known competing financial interests or personal relationships that could have appeared to influence the work reported in this paper.

☐The authors declare the following financial interests/personal relationships which may be considered as potential competing interests:

 Click here to access/download;Supplementary Material;SupplementaryMaterial1.0.docx

Strengthening and failure mechanisms during tension of a Ti-6Al-4V alloy-based nanocomposite processed by laser powder bed fusion

by

B. Guennec, A. Hattal, K. Nagano, A. Hocini, K. Mukhtarova, T. Kinoshita, N. Horikawa, H. Fujiwara, J. Gubicza, M. Djemaï and G. Dirras

Supplementary materials

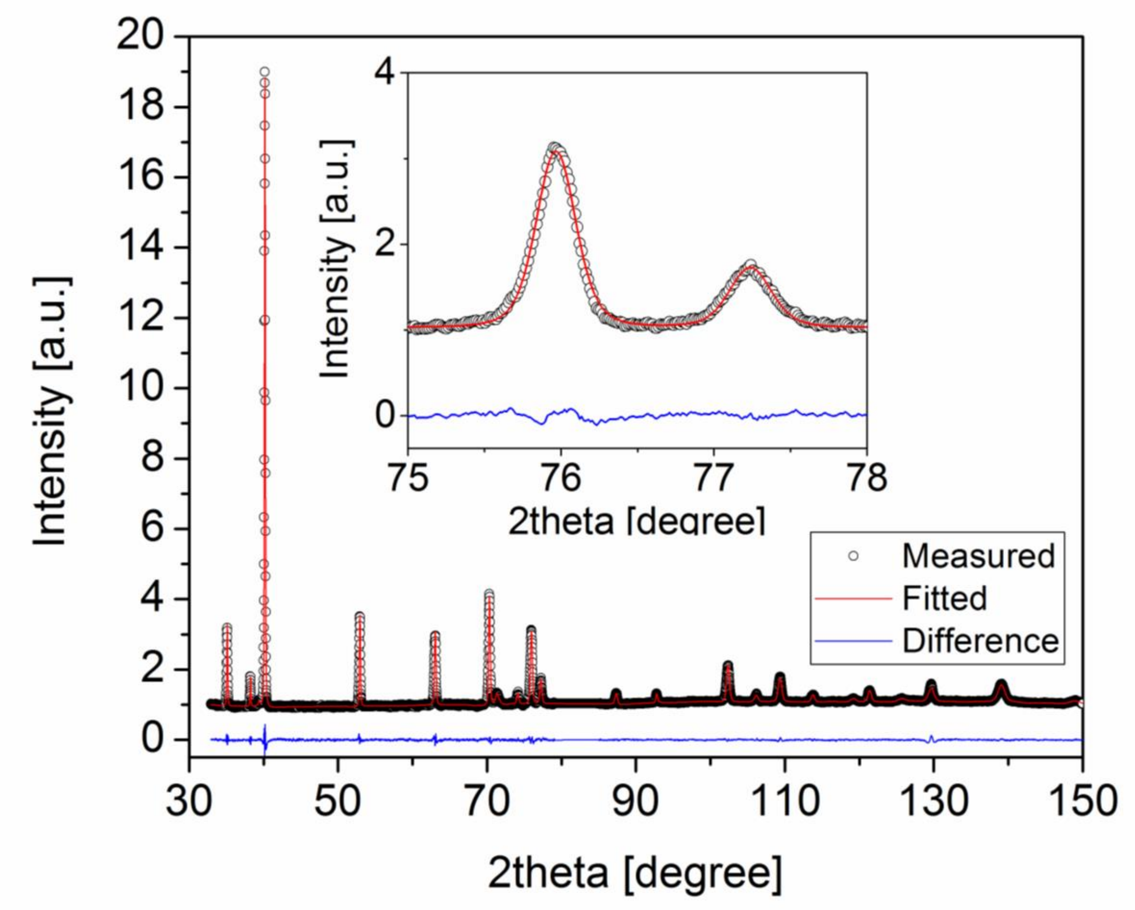

Fig. S1: CMWP fitting for a specimen strained up to 2.25%. The open circles and the solid line represent the measured and the calculated patterns in the case of the best fitting. The difference between the measured and calculated data is shown at the bottom of the figure. A magnified part of the pattern is shown in the inset.

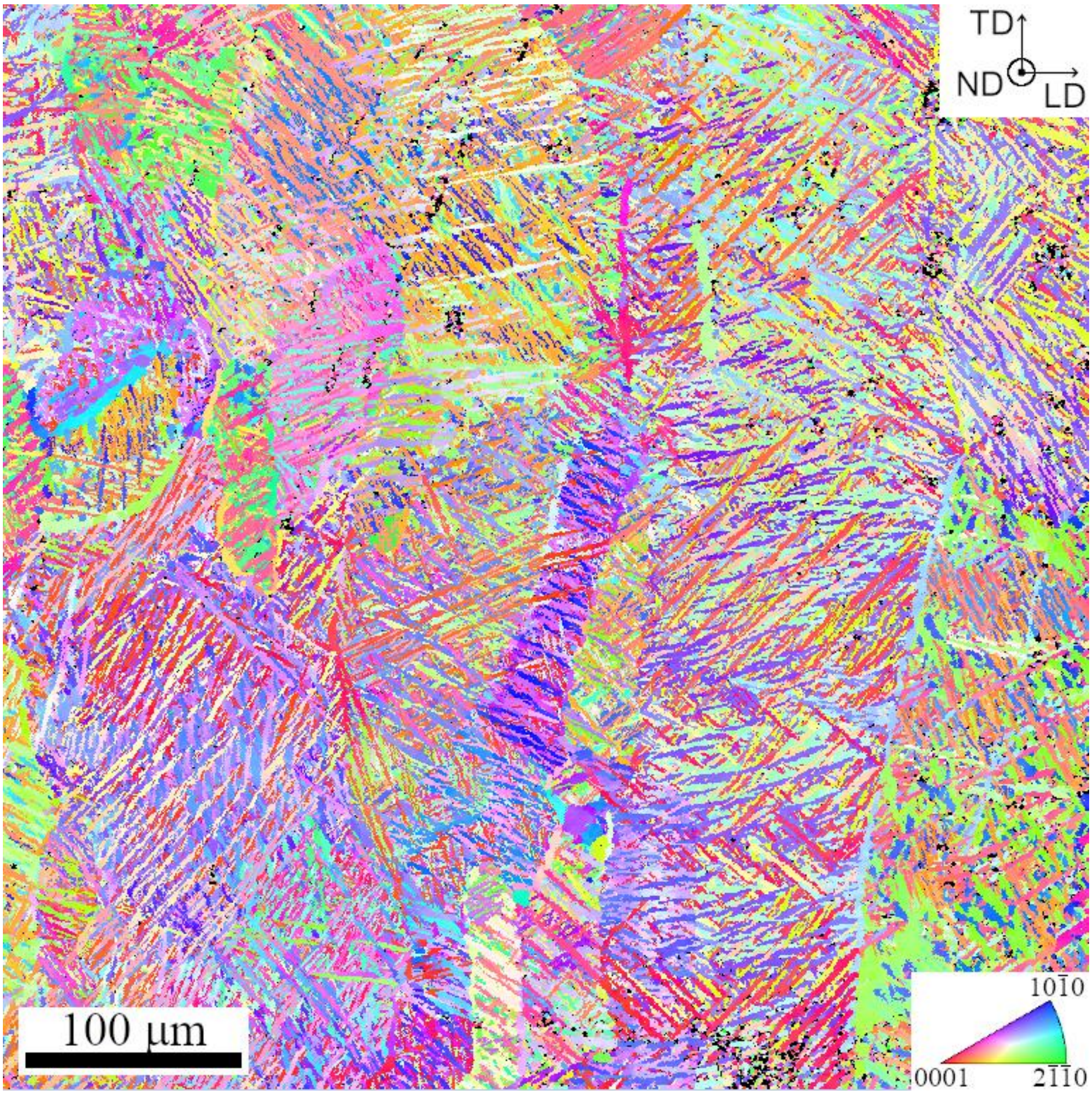

Fig. S2: Ti-α IPF map over a 550 μm × 550 μm region (Step size of 0.70 μm) used as the basis for the statistical approach carried out in Section 4. The horizontal axis corresponds to the actual LD, whereas the vertical axis TD corresponds to BD. Confidence index > 0.100.

Table S1: Analysis of the orientation of the plane from 24 distinct slip systems in Fig. 8(a) and (c). Detected slip system in Fig. 8(a) is underlined. Most plausible activated system(s) in Spot *A*' ~ *E*' of Fig. 8(c) are highlighted by bold typography. (Actual angles measured on the micrographs are: *A*': -37.1°; *B*': -33.5°; *C*': -4.2°; *D*': -6.4°; *E*': 71.9°)

Slip plane	Slip direction	Fig. 8(a)		Fig. 8(c) - Spot *A*'		Fig. 8(c) - Spot *B*'		Fig. 8(c) - Spot *C*'		Fig. 8(c) - Spot *D*'		Fig. 8(c) - Spot *E*'	
		m	θ (°)	*m*	θ (°)	*m*	θ (°)	*m*	θ (°)	*m*	θ (°)	*m*	θ (°)
(0001)	$[11\bar{2}0]$	0.004	-89.1	0.214	71.1	0.216	70.6	0.235	75.5	0.131	72.3	**0.207**	**70.7**
(0001)	$[\bar{2}110]$	0.007	-89.1	0.274	71.1	0.282	70.6	0.104	75.5	0.279	72.3	**0.283**	**70.7**
(0001)	$[1\bar{2}10]$	0.011	-89.1	0.060	71.1	0.066	70.6	0.132	75.5	0.148	72.3	**0.076**	**70.7**
$(10\bar{1}0)$	$[1\bar{2}10]$	0.127	-79.8	0.185	-11.9	0.197	-11.9	**0.436**	**-4.0**	0.411	-25.9	0.227	-10.8
$(1\bar{1}00)$	$[11\bar{2}0]$	0.355	-16.2	**0.452**	**-36.1**	**0.451**	**-36.1**	0.064	-75.9	**0.379**	**-7.2**	0.453	-35.0
$(01\bar{1}0)$	$[\bar{2}110]$	$\underline{0.482}$	$\underline{28.9}$	0.267	42.5	0.254	44.6	0.372	-20.2	0.032	83.4	0.226	50.3
$(10\bar{1}1)$	$[1\bar{2}10]$	0.107	-70.1	0.192	14.0	0.205	14.0	0.447	25.3	0.294	-53.2	0.236	15.0
$(10\bar{1}1)$	$[\overline{21}13]$	0.117	-82.9	0.136	-40.8	0.143	-41.0	0.323	-39.3	0.432	5.4	0.165	-40.4
$(10\bar{1}1)$	$[11\bar{2}\bar{3}]$	0.316	-34.5	0.299	-62.9	0.297	-62.8	0.167	50.1	0.397	20.7	0.303	-61.5
$(10\bar{1}\bar{1})$	$[1\bar{2}10]$	0.312	6.4	0.499	0.2	0.499	-0.3	0.053	85.3	0.274	-40.6	0.497	-0.3
$(10\bar{1}\bar{1})$	$[\bar{2}11\bar{3}]$	0.423	45.8	0.107	-56.2	0.092	-59.0	0.280	-47.8	0.103	58.4	0.067	-64.3
$(10\bar{1}\bar{1})$	$[11\bar{2}3]$	0.429	4.9	0.364	58.4	0.356	59.1	0.377	10.0	0.158	76.3	0.332	61.8
$(\bar{1}101)$	$[11\bar{2}0]$	0.035	-70.1	0.208	14.0	0.203	14.0	0.013	25.3	0.434	-53.2	0.208	15.0
$(\bar{1}101)$	$[1\bar{2}13]$	0.021	-70.1	0.108	14.0	0.096	14.0	0.246	25.3	0.280	-53.2	0.085	15.0
$(\bar{1}101)$	$[\bar{2}11\bar{3}]$	0.036	-82.9	0.499	-40.8	0.498	-41.0	0.257	-39.3	0.214	5.4	0.496	-40.4
$(\bar{1}10\bar{1})$	$[11\bar{2}0]$	0.025	-82.9	0.428	-40.8	0.423	-41.0	0.426	-39.3	0.012	5.4	0.410	-40.4
$(\bar{1}10\bar{1})$	$[1\bar{2}1\bar{3}]$	0.417	-34.5	0.153	-62.9	0.160	-62.8	0.013	50.1	0.014	20.7	0.176	-61.5
$(\bar{1}10\bar{1})$	$[\bar{2}113]$	0.252	-34.5	0.309	-62.9	0.315	-62.8	0.074	50.1	0.221	20.7	0.335	-61.5
$(0\bar{1}11)$	$[\bar{2}110]$	0.427	6.4	0.109	0.2	0.110	-0.3	0.027	85.3	0.313	-40.6	0.099	-0.3
$(0\bar{1}11)$	$[11\bar{2}3]$	0.264	6.4	0.152	0.2	0.151	-0.3	0.001	85.3	0.456	-40.6	0.161	-0.3
$(0\bar{1}11)$	$[1\bar{2}13]$	0.147	45.8	0.074	-56.2	0.064	-59.0	0.467	-47.8	0.001	58.4	0.045	-64.3
$(0\bar{1}1\bar{1})$	$[\bar{2}110]$	0.368	45.8	0.018	-56.2	0.016	-59.0	0.321	-47.8	0.055	58.4	0.010	-64.3
$(0\bar{1}1\bar{1})$	$[11\bar{2}\bar{3}]$	0.136	4.9	0.045	58.4	0.039	59.1	0.263	10.0	0.080	76.3	0.032	61.8
$(0\bar{1}1\bar{1})$	$[1\bar{2}13]$	0.360	4.9	0.145	58.4	0.147	59.1	0.067	10.0	0.003	76.3	0.141	61.8
